# Biosynthesis of the mycotoxin tenuazonic acid by a fungal NRPS–PKS hybrid enzyme

**DOI:** 10.1038/ncomms9758

**Published:** 2015-10-27

**Authors:** Choong-Soo Yun, Takayuki Motoyama, Hiroyuki Osada

**Affiliations:** 1 Chemical Biology Research Group, RIKEN Center for Sustainable Resource Science, 2-1 Hirosawa, Wako-shi, Saitama 351-0198, Japan

## Abstract

Tenuazonic acid (TeA) is a well-known mycotoxin produced by various plant pathogenic fungi. However, its biosynthetic gene has been unknown to date. Here we identify the TeA biosynthetic gene from *Magnaporthe oryzae* by finding two TeA-inducing conditions of a low-producing strain. We demonstrate that TeA is synthesized from isoleucine and acetoacetyl-coenzyme A by TeA synthetase 1 (TAS1). TAS1 is a unique non-ribosomal peptide synthetase and polyketide synthase (NRPS–PKS) hybrid enzyme that begins with an NRPS module. In contrast to other NRPS/PKS hybrid enzymes, the PKS portion of TAS1 has only a ketosynthase (KS) domain and this domain is indispensable for TAS1 activity. Phylogenetic analysis classifies this KS domain as an independent clade close to type I PKS KS domain. We demonstrate that the TAS1 KS domain conducts the final cyclization step for TeA release. These results indicate that TAS1 is a unique type of NRPS–PKS hybrid enzyme.

## Abstract (web summary)

Tenuazonic acid is a mycotoxin produced by various plant pathogenic fungi but its biosynthetic gene is unknown to date. Here, the authors identify the tenuazonic acid biosynthetic gene encoding a protein with a unique KS domain that conducts cyclization step for tenuazonic acid release in *Magnaporthe oryzae*.

## Graphical abstract

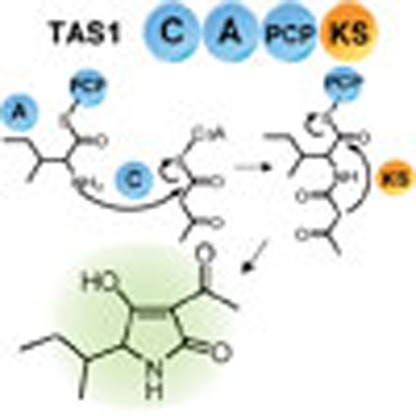

The mycotoxin tenuazonic acid (TeA), (5*S*)-3-acetyl-1,5-dihydro-4-hydroxy-5-[(1*S*)-1-methylpropyl]-2*H*-pyrrol-2-one, is a tetramic acid derivative first isolated from the broth of an *Alternaria tenius* culture^1^. *Alternaria* is a ubiquitous plant pathogenic fungus that causes both spoilage of various food crops and fruits in the field and post-harvest decay^2^, and TeA has been detected in various *Alternaria*-contaminated crops, fruits and vegetables^3^,^4^,^5^. In addition, the plant pathogenic fungi *Phoma sorghina* (a pathogen of sorghum) and *Magnaporthe oryzae* (a pathogen of rice) reportedly produce TeA^6^,^7^. TeA is the most toxic of the *Alternaria* toxins. It inhibits protein biosynthesis on ribosomes by suppressing the release of new protein^8^. It also reportedly shows biological properties including antitumor, antibacterial, antiviral and phytotoxic activities^9^,^10^,^11^.

TeA is thought to be a hybrid of an isoleucine and two acetates^12^. Due to its tetramic acid-containing structure (Fig. 1a), TeA is also expected to be a product of a polyketide synthase and non-ribosomal peptide synthetase (PKS–NRPS) hybrid enzyme^13^.

Typical fungal secondary metabolite compounds polyketides and non-ribosomal peptides are synthesized by PKSs and NRPSs, respectively. There are two types of fungal PKSs. One is iterative type I PKSs, consisting of multiple catalytic domains that contain ketosynthase (KS), acyltransferase (AT) and acyl carrier protein (ACP) main domains, along with several optional β-keto processing domains, such as β-ketoacyl reductase (KR), dehydratase (DH) and trans-acting enoyl reductase domains in single enzyme^14^. According to the absence or presence of β-keto processing domains, iterative type I PKSs can be subdivided into non-reducing (NR-), partially reducing (PR-) and highly reducing (HR-) PKSs^15^,^16^. The other type is type III PKSs, consisting of a homodimeric KS^17^. Type II PKSs, which include several individual enzymes^18^, have to date only been identified in bacteria.

In addition, fungal PKS–NRPS hybrid enzymes, whose structure consists of an iterative type I PKS followed by a single module NRPS, produce a wide variety of structurally diverse secondary metabolites including polyketide-amino acid hybrids^19^,^20^,^21^,^22^,^23^. In the typical fungal PKS–NRPS, the PKS portion consists of KS, AT and ACP domains, along with several modifying domains such as KR, DH and methyltransferase domains. The NRPS portion consists of adenylation (A), thiolation (T, also known as PCP for peptidyl carrier protein), condensation (C) and terminal release or cyclization (R, reductase or DKC, Dieckmann cyclization) domains^18^. Similar types of hybrid enzyme have been observed in bacteria^24^,^25^. In contrast to PKS–NRPS, NRPS–PKS hybrid enzymes (which begin with a NRPS module) also exist; however, to date, this type of enzyme has only been found in bacteria^26^,^27^,^28^,^29^,^30^,^31^ (Supplementary Fig. 1).

Herein, we describe the identification of the TeA biosynthetic gene and the functional *in vivo* and *in vitro* analysis of TeA synthetase 1 (TAS1) in *M. oryzae*. TAS1 is a unique type of NRPS–PKS hybrid enzyme of fungal origin. In addition, we discuss the unique domain structure of TAS1 and demonstrate that its KS domain is indispensable for TeA biosynthesis.

## Results

### Identification of the TeA biosynthetic gene

**Figure 1:**
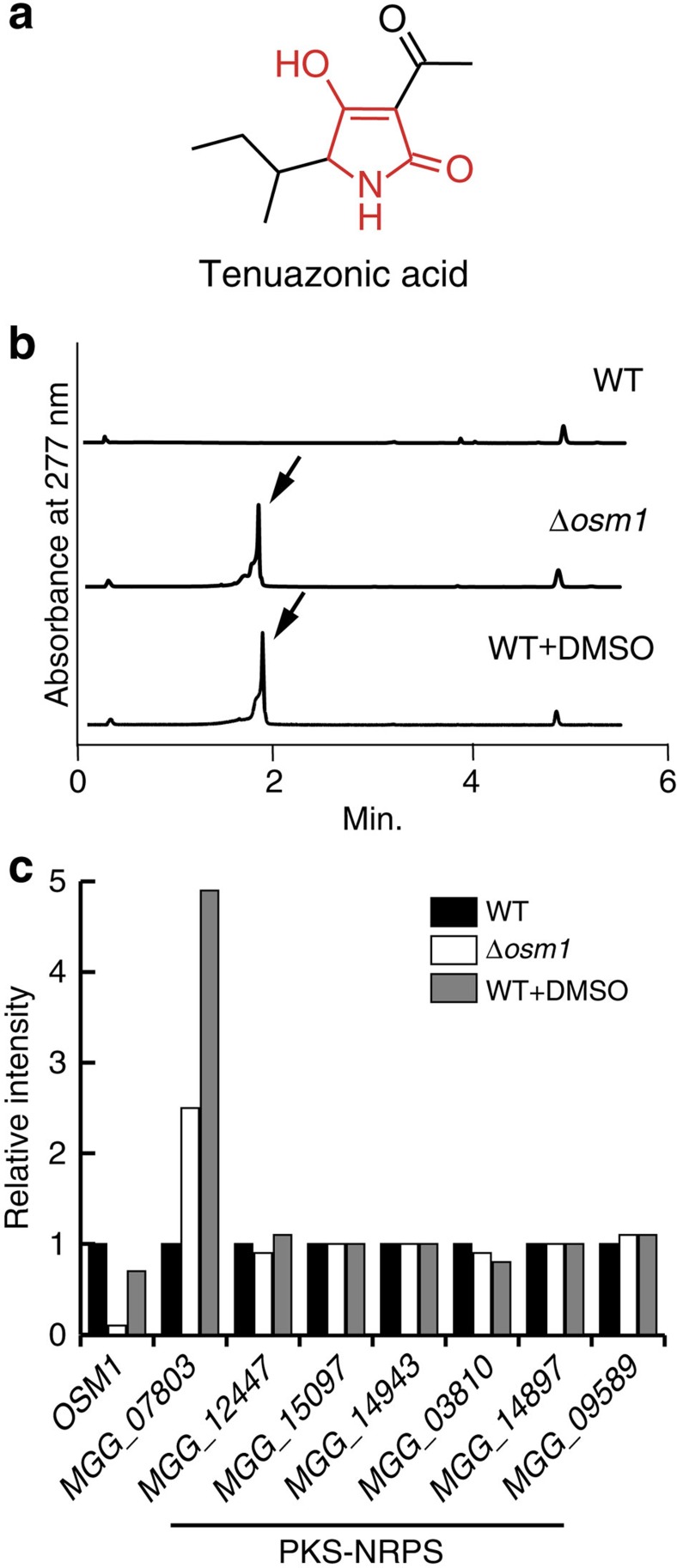
Tenuazonic acid biosynthetic gene identification. (**a**) Chemical structure of TeA. Red portions indicate the tetramic acid structure. (**b**) UPLC analysis of metabolites extracted from strains of *M. oryzae* Kita 1 (wild type (WT)), *Δosm1* and DMSO-added culture conditions. Each strain was static-cultured for 5 days at 25 °C. Arrows indicate TeA peak. (**c**) Signal intensities of *OSM1*, *MGG_07803* and six PKS–NRPS genes in a microarray analysis with WT (black), *Δosm1* (white) and strains cultivated under DMSO-added culture conditions (grey).

Secondary metabolite production in microorganisms is regulated by environmental conditions for adaptation. For example, OSM1, a Hog1-related mitogen-activated protein kinase in *M. oryzae*, is reportedly involved in the response to environmental signals such as hyperosmotic stress^32^. Therefore, we hypothesized that OSM1 is also involved in secondary metabolism regulation in *M. oryzae*. To confirm this hypothesis, we used *M. oryzae* to construct *OSM1* (*MGG_01822*) knockout strains (Δ*osm1*; Supplementary Methods and Supplementary Fig. 2) and analysed their metabolites with ultra performance liquid chromatography/mass spectroscopy (UPLC/MS). The production of one metabolite was induced in the Δ*osm1* strains under static culture conditions (Fig. 1b). We then obtained the ultraviolet pattern and mass spectra of the induced metabolite, which had a molecular mass of 197.2 (*m/z*=196.2 [M-H]^−^) corresponding to the fungal secondary metabolite TeA (Fig. 1a, Supplementary Fig. 3). Although a previous report described the production of TeA in *M. oryzae*, the *M. oryzae* Kita 1 strain used in this study did not produce a detectable amount of TeA under our laboratory culture conditions (Methods section). In addition, while screening compounds that induce the production of secondary metabolites, we found that 1% dimethylsulphoxide (DMSO) also induced TeA production (14 mg l^−1^) in *M. oryzae* under static culture conditions (Fig. 1b). Although a number of studies have detected TeA in plants infected with TeA-producing fungi^3^,^4^,^5^, the biosynthetic gene of this compound has been unknown to date. Thus, we undertook this study to identify the biosynthetic gene of TeA in *M. oryzae*. Two TeA-inducing culture conditions enabled us to explore the TeA biosynthetic gene in this organism.

To identify the TeA biosynthetic gene, we performed a custom DNA microarray analysis using total RNA of *M. oryzae* extracted under the two TeA-inducing culture conditions and non-inducing normal culture conditions. TeA is expected to be the product of a PKS–NRPS hybrid enzyme^13^, and the genome sequence of *M. oryzae*^33^ has six genes encoding such PKS–NRPS enzymes. However, the transcriptional levels of these genes were unchanged under TeA-inducing conditions. Of the remaining secondary metabolism-related genes, only one gene (*MGG_07803*) was found to be commonly upregulated (by more than twofold) under the two TeA-inducing conditions (Fig. 1c, Supplementary Table 1), suggesting that it is involved in TeA biosynthesis.

### Confirmation of the TeA biosynthetic gene

**Figure 2:**
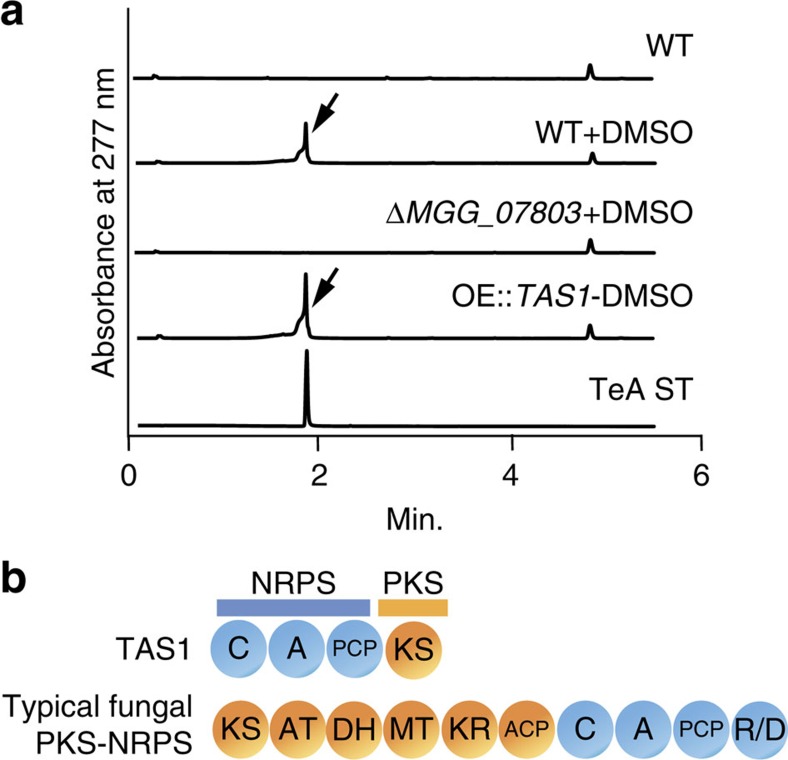
Confirmation of the TeA biosynthetic gene and domain structure. (**a**) UPLC analysis of metabolites extracted from WT, WT+DMSO, *ΔMGG_07803*+DMSO and a *TAS1* overexpression strain (OE::*TAS1*) without DMSO. Each strain was static-cultured for 5 days at 25 °C. Arrows indicate TeA peak. A 100 μM TeA standard (TeA ST) was also analysed. (**b**) Domain structure of TAS1 and typical fungal PKS–NRPS hybrid enzyme; blue indicate the NRPS portion, and yellow indicates the PKS portion.

To ensure that *MGG_07803* is the biosynthetic gene of TeA, we constructed an *MGG_07803* knockout. The knockout strain lost the capacity to produce TeA under the DMSO-added culture condition (Fig. 2a, Supplementary Fig. 4), confirming that this gene is the TeA biosynthetic gene. Accordingly, we renamed *MGG_07803* to *TAS1*. Because *TAS1* is automatically annotated from the genome sequence of *M. oryzae*, we conducted rapid amplification of complementary DNA (cDNA) ends-polymerase chain reaction with the cDNA of *M. oryzae* that was cultured with 1% DMSO and identified an open reading frame (ORF) region (Supplementary Methods and Supplementary Fig. 5). Using this ORF, we constructed *TAS1* overexpression strains by inserting the *Aspergillus oryzae TEF1* promoter into the upstream of the *TAS1* start codon via homologous recombination. UPLC/MS analysis of extracted metabolites from the resulting strain showed 28 mg l^−1^ TeA-producing capability without DMSO addition (Fig. 2a, Supplementary Fig. 4). This outcome also supported the conclusion that TAS1 is the TeA biosynthetic enzyme. *TAS1* encodes an NRPS–PKS hybrid protein of 1,602 amino acids that consists of C, A and PCP domains in the NRPS portion and a KS domain in the PKS portion (Fig. 2b). The PKS portion of this domain structure is distinct from that of typical fungal PKS–NRPS hybrid enzymes with type I PKSs (Fig. 2b). Intriguingly, the NRPS portion of TAS1 also lacks the terminal release or cyclization domain (R or DKC)^20^, required for product release from the enzyme and generally present in fungal PKS–NRPS hybrid enzymes. In contrast, similar to type III PKSs, the PKS portion of TAS1 has only a KS domain. However, it shows very low similarity to known type III PKSs. Moreover, fungal PKS–NRPS hybrid enzymes have been reported but there are no reports of NRPS–PKS type fungal hybrid enzymes, even though bacterial NRPS–PKS enzymes have been identified. Therefore, TAS1 is a novel type of fungal secondary metabolite biosynthetic enzyme.

### Substrate specificity of TAS1

**Figure 3:**
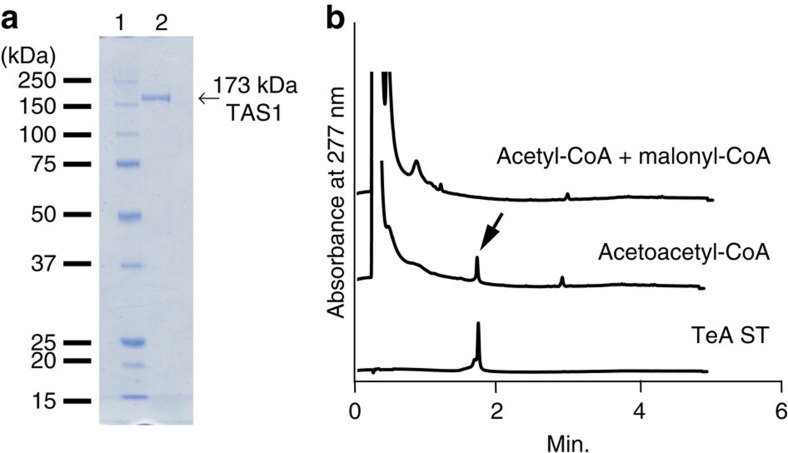
Recombinant TAS1 expression and substrate specificity. (**a**) Purified C-terminally 8 × His-tagged TAS1 protein was loaded and analysed with a 5–20% sodium dodecyl sulphate-polyacrylamide gel electrophoresis gradient gel. Line 1, molecular mass markers. Line 2, purified TAS1 (500 ng). (**b**) UPLC analysis of the products of the TAS1 enzyme reaction with acetoacetyl-CoA and a mixture of acetyl-CoA and malonyl-CoA. Arrows indicate TeA peak. A 100 μM TeA standard (TeA ST) was also analysed. *In vitro* reaction conditions are described in the Methods section.

On the basis of the structure of TeA, we expected that isoleucine and diketide are necessary for TeA biosynthesis. As TAS1 is a novel fungal NRPS–PKS enzyme with a PKS portion that has low similarity to that of type III PKSs, it is unknown whether this diketide is synthesized by TAS1. If TAS1 can synthesize this diketide, TeA can be synthesized by a single enzyme, TAS1, from isoleucine, acetyl-coenzyme A (CoA) and malonyl-CoA. Alternatively, TAS1 might use acetoacetyl-CoA as a source of diketide. To clarify this point, we prepared a recombinant enzyme and analysed its substrate specificity *in vitro*. The 173-kDa TAS1 protein was overexpressed in a protease-deficient yeast strain BJ5464 using *Saccharomyces cerevisiae* 2 μm episomal vector carrying the genes-encoding TAS1 under the control of the *ADH1* promoter. To obtain the active form of TAS1, the 4′-phosphopantetheinyl transferase (PPTase; MGG_17878) of *M. oryzae*, which catalyses the essential post-translational activation of carrier proteins of NRPS and PKS, was coexpressed as a non-tagged protein. The purified C-terminally His-tagged recombinant TAS1 protein synthesized TeA as a single enzyme from isoleucine with acetoacetyl-CoA, not acetyl-CoA and malonyl-CoA (Fig. 3a,b), which clearly indicated that TAS1 is the TeA biosynthetic enzyme. The *k*_cat_ was determined to be (14.9±0.3) × 10^−3^ min^−1^, and the *K*_M(app)_ was (44.3±3.1) × 10^−2^ mM for isoleucine. For acetoacetyl-CoA, the *k*_cat_ was determined to be (13.1±0.2) × 10^−3^ min^−1^, and the *K*_M(app)_ was (1.2±0.1) × 10^−2^ mM. For ATP, the *k*_cat_ was determined to be (15.3±0.2) × 10^−3^ min^−1^, and the *K*_M(app)_ was (8.1±0.5) × 10^−2^ mM (Supplementary Fig. 6).

### Homologue search and phylogenetic analysis

To investigate the distribution of this type of hybrid protein, we conducted a Basic Local Alignment Search Tool (BLAST) search for proteins with sequences similar to that of TAS1 via the National Center for Biotechnology Information (http://www.ncbi.nim.nih.gov) and *Alternaria* genomes database (http://alternaria.vbi.vt.edu)^34^. *Alternaria* species are known to be major TeA producers. Homologue proteins that share 50% amino acid identity with TAS1 were found in *Alternaria* genomes database and their domain structures were identical to that of TAS1 (C–A–PCP–KS). TAS1 homologue proteins in *Alternaria* spp. were highly conserved and share over 97% identity. In addition, proteins with domain structures identical to that of TAS1 were also found in other fungal species from the National Center for Biotechnology Information database, which suggests that this type of hybrid enzyme is distributed in Ascomycota and Basidiomycota fungi (Supplementary Table 2).

**Figure 4:**
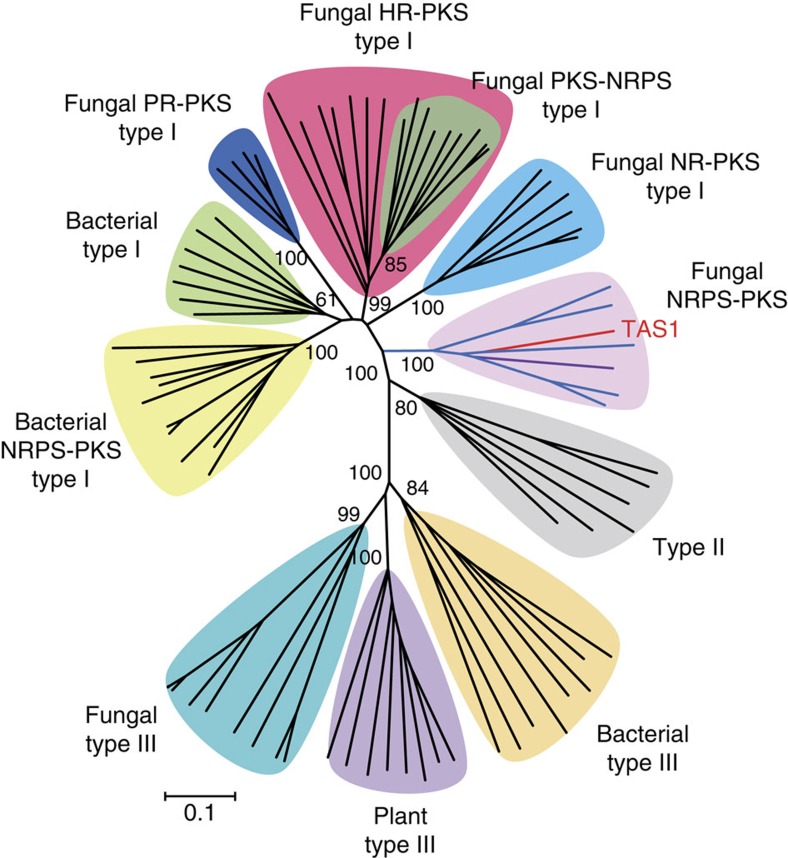
Phylogenetic analysis of the KS domain in TAS1. Phylogenetic analysis of the TAS1 KS domain and other KS domains. Domains were aligned with MUSCLE, and the tree was constructed with the neighbour-joining method. Scale bar, 0.1 substitutions per site. The TAS1 KS domain is indicted with a red line and purple line indicates TAS1 homologue of *A. alternate*. The KS domains of other TAS1 homologues are indicated with blue lines.

However, similar protein sequences matched with TAS1 from bacterial sources had domain structures that differed considerably from that of TAS1. These proteins had a typical PKS structures consisted of KS, AT and ACP domains (Supplementary Table 2) at the end of the NRPS. This difference suggests that the C–A–PCP–KS domain structure of the NRPS–PKS hybrid enzyme is specific to fungi. We also conducted phylogenetic analysis of the KS domain in TAS1 with other KS domains from types I, II, III PKSs and PKS–NRPS hybrid proteins including the top five protein sequences from fungal and bacterial sources extracted from the TAS1 BLAST search result (Supplementary Table 2). Despite the single KS domain structure of the PKS portion, the KS domain of TAS1 was not classified as a type III PKS (Fig. 4, Supplementary Fig. 7). In addition, the KS domains from fungal PKS–NRPS hybrid enzymes were classified into one independent clade; however, the KS domain of TAS1 was not classified therein. Interestingly, protein sequences similar to TAS1 in bacteria were classified into an independent bacterial clade of type I PKSs with other proteins that reportedly interact with NRPS modules, including NRPS–PKS hybrid proteins^31^. In contrast, the KS domain of TAS1 formed a new clade with the homologues from fungal sources, which was close to the type I PKSs (Fig. 4, Supplementary Fig. 7). This outcome indicates that TAS1 is a unique type of NRPS–PKS hybrid enzyme.

To further analyse TAS1 homologues, we conducted phylogenetic analysis of all KS domains, except for three homologues that have a truncated C-terminal KS domain (Supplementary Table 2), extracted from BLAST search results. All KS domains from TAS1 homologues were also classified into an independent clade different from type I PKS clades (Supplementary Fig. 8). In addition, TAS1 homologues were divided into four sub-clades. Sub-clade A has five KS domains and includes the TAS1 from *M. oryzae* and a TAS1 homologue from *A. alternata*. These two strains were already known to produce TeA. This suggests that strains have a TAS1 homologue in the sub-clade A are TeA producer. Interestingly, sub-clade B and C consists only of homologues from Basidiomycota, whereas sub-clade D mainly contains homologues from fungi that infect insects, except for *Fusarium* spp.

### Functional analysis of the TAS1 KS domain

**Figure 5:**
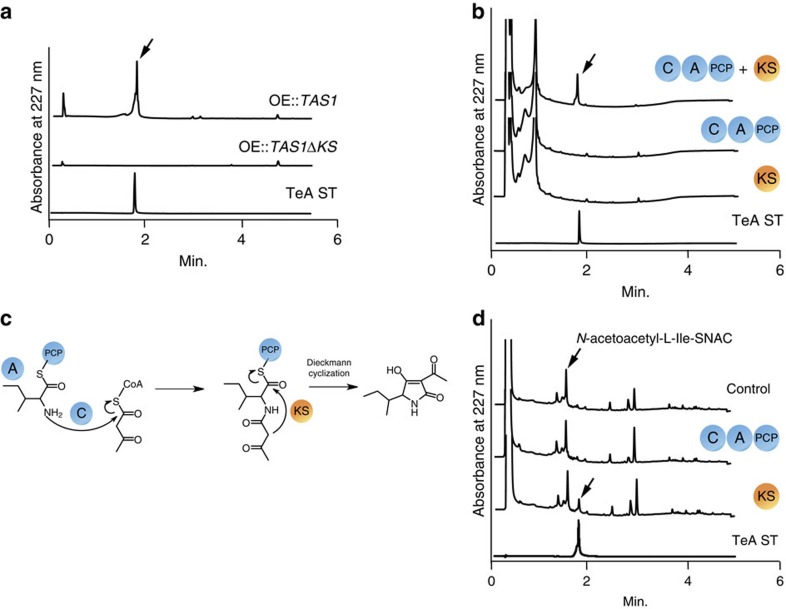
KS domain analysis. (**a**) UPLC analysis of metabolites extracted from *TAS1* overexpression (OE::*TAS1)* and KS domain-disrupted *TAS1* overexpression (OE::*TAS1ΔKS)* strains. Arrows indicate TeA peak. A 100 μM TeA standard (TeA ST) was also analysed. (**b**) UPLC analysis of enzyme reaction products. C-A-PCP domain proteins, KS domain proteins or both of TAS1 were used as enzymes. A 100 μM TeA ST was also analysed. *In vitro* reaction conditions are described in the Methods section. Arrow indicates TeA peak. (**c**) Proposed model for TeA biosynthesis. (**d**) UPLC analysis of enzyme reaction products. Soluble C–A–PCP domain proteins and KS domain proteins were used to investigate cyclization capability with the artificial intermediate *N*-acetoacetyl-L-Ile-SNAC. A control reaction was conducted with cell-free-synthesized soluble protein without messenger RNA. *In vitro* reaction conditions are described in the Methods section. Arrow indicates TeA peak. SNAC, *N*-acetylcysteamine.

Generally, the KS domain in the PKS enzyme is responsible for the control of polyketide product chain length via ketide extension. However, TAS1 uses acetoacetyl-CoA as a source of diketide to produce TeA, which means that ketide extension to yield the diketide (acetoacetyl-CoA) is unnecessary for TeA synthesis. Thus, we investigated whether the KS domain of TAS1 is necessary for TeA biosynthesis. We constructed the KS domain deleted *TAS1* overexpression strains and found that the constructed strains lost the capability to produce TeA (Fig. 5a, Supplementary Fig. 9). This result showed that the KS domain is necessary for TeA biosynthesis. For further confirmation, we prepared separately expressed recombinant proteins with the C–A–PCP and KS domain portions of TAS1 by using a cell-free protein synthesis system (Supplementary Fig. 10a). Analysis of the TeA biosynthetic capability of these proteins showed that TeA was produced only when the C–A–PCP domains and the KS domain coexisted, which clearly indicated that the KS domain of TAS1 is indispensable for TeA biosynthesis (Fig. 5b). However, the exact role of the KS domain in TeA biosynthesis remains unknown.

From the perspective of the domain structure of TAS1, we proposed a model for TeA biosynthesis and hypothesized that the final cyclization step in TeA production is conducted by the KS domain (Fig. 5c). To support this hypothesis, we synthesized an artificial intermediate of TeA biosynthesis using *N*-acetylcysteamine (SNAC), *N*-acetoacetyl-L-Ile-SNAC (Supplementary Methods and Supplementary Fig. 11), to analyse the cyclization capacity of the KS domain with the KS and C–A–PCP domain proteins synthesized with the cell-free system. The results showed that only the KS domain protein produced TeA, which indicated that this TAS1 domain is responsible for the final cyclization step in TeA production (Fig. 5d). In addition, we confirmed the condensation capacity of activated isoleucine and acetoacetyl-CoA by C–A–PCP or KS domain proteins using L-Ile-SNAC (Supplementary Fig. 11). The results showed that the condensed product (*N*-acetoacetyl-L-Ile-SNAC) is only produced by the C–A–PCP domain protein from L-Ile-SNAC and acetoacetyl-CoA, which indicated that the condensation reaction takes place via the C domain rather than the KS domain in TAS1 (Supplementary Fig. 12).

## Discussion

The recent sequencing of fungal genomes has shown that fungi are richer in secondary metabolism-related genes than once believed. However, the majority of these genes are silent or quiescent under laboratory culture conditions^35^. During the past decade, successful approaches such as epigenetic regulation, promoter exchange and heterologous expression have been applied to activate these silent gene clusters in fungi^36^,^37^,^38^,^39^. In this study, we activated the TeA biosynthetic gene in *M. oryzae* via disruption of *OSM1*, showing the possibility that disruption of an environmental signal response system can influence some of the poorly expressed or silent secondary metabolite genes in *M. oryzae*.

TeA was first reported in 1957 by Rosett *et al.*^1^; however, the TeA biosynthetic gene was not identified in the nearly 60 years that followed despite many reports of TeA detection. The chemical structure of TeA suggested that TeA is synthesized by a PKS and NRPS hybrid enzyme, and fungal PKS–NRPS hybrid enzyme was considered as biosynthetic enzyme of TeA, because reports of NRPS–PKS type fungal enzymes were not forthcoming. The TAS1 that we have identified is the first reported fungal NRPS–PKS enzyme, which may explain why the TeA biosynthetic gene has remained unidentified for so long. Our result also shows the limitations of the *in silico* prediction of polyketide and non-ribosomal peptide biosynthetic pathways from genomic sequence data.

The KS domain of TAS1 is quite different from other KS domains. Phylogenetic position of the KS domain of TAS1 is nearest to PKS type I. However, it does not belong to any clade of fungal NR-, PR- or HR-PKS type I and is clearly separated from the clades of PKS type I KS domains (Supplementary Fig. 7). In contrast to fungal KS domains of PKS type I, the KS domain of TAS1 is non-iterative. Moreover, the conserved catalytic triad residues of the KS domain in TAS1 is different from that of type I PKSs (Cys–His–His) and identical to that of type III PKSs (Cys–His–Asn; Supplementary Fig. 13)^17^,^40^. The catalytic triads of fungal homologues of TAS1 are also identical to that of TAS1. However, the residues surrounding the catalytic triad of TAS1 are more frequently conserved in the KS domains of type I PKSs than in those of type III PKSs (Supplementary Fig. 13). According to the above data, we suggest that the KS domain of TAS1 can be classified into a new group different from KS domains of type I, II, and III PKSs.

TAS1 consists of an NRPS and a unique type PKS KS domain involved in cyclization and product release. Cyclization and product release generally take place via the thioesterase (TE) or Claisen cyclase domains in fungal type I NR-PKSs. In contrast, there is no fused TE domain in most HR-PKSs and product release and cyclization require an in trans interaction with separate enzymes^41^,^42^. In addition, the cyclization step of macrocyclic peptides that produced by fungal NRPSs is mainly conducted by a terminal condensation-like domain^43^. Interestingly, there are two product release mechanisms in fungal PKS–NRPS hybrid enzymes^20^, which differ from the single PKS or NRPS. The first is the final R domain mediated release mechanism. The R domain is thought to catalyse the reductive release from the sequence similarities to short-chain dehydrogenase/reductase family proteins. The second mechanism is DKC, catalysed by the terminal domain (D). Extensive investigations of tenellin, fusaridione and α-cyclopiazonic acid (fungal PKS–NRPS products) biosynthesis suggested that the formation of tetramic acid derivatives via DKC is the product release mechanism^44^,^45^,^46^. In the proposed model for TeA biosynthesis (Fig. 5c), the final TeA release mechanism is expected to be DKC. However, TAS1 does not contain any of these cyclization and product release related domains. In contrast, type III PKSs have only a KS domain and the elongation/cyclization step for polyketide production is conducted with a single enzyme. In addition, biosynthesis analysis of the polyketide RK-682, which contains tetronic acid (an analogue of tetramic acid), showed that a KS-like enzyme (RkD) responsible for assembly of the tetronic acid moiety by catalysing intermolecular C–C and C–O bond formation^47^. In other polyketides-containing tetronic acid—chlorothricin and quartromicin—biosynthetic pathways also contain these type of KS-like enzymes^48^,^49^. However, tetramic acid ring formation in TeA occurs via an intramolecular reaction catalysed by a KS domain (Dieckmann cyclization).

To study the peptide cyclization capability of TE domain in bacterial NRPSs, SNAC derivatives were extensively used as artificial intermediates of the enzyme reaction^50^,^51^. SNAC mimics the enzyme-phosphopantetheine moiety that carries enzymatic intermediates in the cyclization enzyme reaction. However, non-enzymatic self-cyclization and non-reactiveness in SNAC derivatives have also been reported^52^,^53^. We synthesized *N*-acetoacetyl-L-Ile-SNAC as an intermediate of TeA biosynthesis to investigate the cyclization capability of the KS domain in TAS1; however, synthesized *N*-acetoacetyl-L-Ile-SNAC showed self-cyclization, the rate of which increased with increasing pH. Thus, we conducted the enzyme reaction at pH 6.5 to minimize self-cyclization and demonstrated that the KS domain in TAS1 is responsible for the final step of cyclization in TeA production.

In fungal type I PKSs, the KS domain generally recognizes acetyl-CoA as a starter unit to produce polyketide. However, the TAS1 KS domain recognizes acetoacetyl-L-Ile hybrid to initiate the cyclization reaction to produce TeA. Acetoacetyl-CoA is an intermediate of the mevalonate pathway and some type III PKS enzymes also recognize acetoacetyl-CoA as a starter unit to produce polyketides^54^,^55^.

The Cys–His–Asn catalytic triad that is conserved in the type III PKSs and KS domain of TAS1 may be involved in cyclization reaction and substrate recognition as in type III PKSs. However, at this time we cannot explain the exact cyclization or substrate recognition mechanisms of the TAS1 KS domain for TeA biosynthesis. Further studies such as crystal structure analysis will be required to clarify this mechanism.

In summary, we identified a gene (*TAS1*) for mycotoxin TeA biosynthesis in *M. oryzae.* We also demonstrated that TAS1 biosynthesizes TeA from isoleucine and acetoacetyl-CoA. TAS1 is the first fungal NRPS–PKS hybrid enzyme identified that consists of the unique domain structure described. TeA-producing plant pathogenic fungi are ubiquitous, and TeA-contaminated crops have attracted increasing public health attention in recent years. We expect the results of this study to stimulate additional investigations of TeA biosynthesis and production regulation mechanisms in pathogenic fungi and shed light on TeA contamination. Moreover, because the unique domain structure of the PKS portion of TAS1 was previously unreported, the results of our study will also contribute to research of PKSs.

## Methods

### Chemicals

Isoleucine, CoA, acetoacetyl-CoA, acetyl-CoA, malonyl-CoA, ATP and TeA were purchased from Sigma-Aldrich (St Louis, MO, USA). Kanamycin (Km), carbenicillin and hygromycin B were purchased from Nacalai (Kyoto, Japan). Blasticidin S was purchased from Funakoshi (Tokyo, Japan). All other reagents were of analytical grade.

### Strains and culture conditions

The strains and plasmids used in this study are listed in Supplementary Table 3. The *M. oryzae* strain kita 1, which is pathogenic in rice plants, was used as a wild-type strain. *M. oryzae* was grown on OMA plates (5% oatmeal agar, Difco, Detroit, MI, USA) or PDA plates (3.9% potato dextrose agar, Difco) at 25 °C. For static culture, *M. oryzae* was grown in 100 μl of liquid YG media (0.5% yeast extract, 2% glucose) on a 96-well flat bottom plate (Iwaki, Tokyo, Japan) at 25 °C without agitation for 5 days. *M. oryzae* was transformed with the *Agrobacterium tumefaciens*-mediated transformation method^56^,^57^. *A. tumefaciens* strain C58 was cultured with a conidial suspension of *M. oryzae* prepared as described previously^58^. Transformants were selected with 500 μg ml^−1^ hygromycin B or 150 μg ml^−1^ blasticidin S when culturing *M. oryzae* transformants. *Escherichia coli* DH5α was grown in Lysogeny broth (LB) at 37 °C, and transformation was performed with a standard method^59^. Km or carbenicillin (50 μg ml^−1^) was added to the *E. coli*-transformant selective medium and solidified with 2% agar for plate culture if needed.

### DNA manipulation

DNA isolation and manipulation were performed with a standard method^59^. Genomic DNA was isolated from *M. oryzae* with a DNeasy plant total DNA isolation kit (Qiagen, Valencia, CA, USA). DNA fragments were isolated from agarose with a gel extraction kit (Qiagen). Plasmids were purified with a QIAprep Spin Miniprep kit (Qiagen). PCR was carried out with a DNAEngine Peltier thermal cycler (Bio-Rad, Hercules, CA, USA). DNA amplification via PCR was conducted with KOD-Plus-neo DNA polymerase (Toyobo, Osaka, Japan). DNA fragment ligation and circularization for vector construction was conducted via infusion reaction using an In-Fusion HD cloning system (Clontech, Palo Alto, CA, USA). DNA sequencing was carried out with a BigDye terminator ver3.1 kit (Applied Biosystems, Foster City, CA, USA). Sequencing products were run on an automated 3730 × l capillary DNA analyzer (Applied Biosystems).

### *MGG_07803* gene disruption

*MGG_07803* was disrupted by exchanging a whole-gene ORF with the hygromycin B resistance gene expression unit via homologous recombination. *MGG_07803* gene disruptants were constructed as follows. Two kb of the upstream region of *MGG_07803* was amplified from the genomic DNA of *M. oryzae* via PCR with the primers ΔM07803_UP-F and ΔM07803_UP-R (fragment 1). The hygromycin B resistance gene expression unit was amplified via PCR from pCSN45 (ref. 60) with primers 5HPH and 3HPH (fragment 2). Two kb of the downstream region of *MGG_07803* was amplified from the genomic DNA of *M. oryzae* via PCR with the primers ΔM07803_DN-F and ΔM07803_DN-R (fragment 3). The vector sequence of pBI121 (Clontech) between the right and left borders was amplified from pBI121 via PCR with primers pBI121-RB and pBI121-LB (fragment 4).

All fragments were gel purified and cloned by using the In-Fusion cloning technology to yield pBI-M07803::HPH. The In-Fusion reaction mixtures were used to transform *E. coli* DH5α, and transformants were selected with Km (50 μg ml^−1^). After the DNA sequences were verified, plasmid DNA containing the correct insert was transformed into *A. tumefaciens*, and the transformants were used for *A. tumefaciens*-mediated transformation. *MGG_07803* disruptants were selected via PCR with primers ΔM07803_CHK_F and ΔM07803_CHK_R that hybridize outside of the upstream and downstream regions of *MGG_07803*, which was used for vector construction. This primer set can amplify 10 kb of the *MGG_07803* ORF, including 2 kb of the up- and downstream regions from wild-type strains, the 5.3-kb hygromycin B resistance gene expression unit, and the up- and downstream regions of *MGG_0780*3 from disruptants (Supplementary Fig. 4). The primers used are listed in Supplementary Table 4.

### *TAS1* gene overexpression

*TAS1* gene overexpression strains were constructed as follows. Two kb of the upstream region of *TAS1* was amplified from the genomic DNA of *M. oryzae* via PCR using primers OETAS1_UP-F and OETAS1_UP-R (fragment 1). The blasticidin S resistance gene expression unit was amplified from pBF101-Δ*Not*I (ref. 58) via PCR with primers 5HPH and 3HPH (fragment 2). The *TEF1* gene promoter region of *A. oryzae* was amplified from *A. oryzae* genomic DNA via PCR with primers AoTEF1-F and AoTEF1-R (fragment 3). The *TAS1* ORF was amplified from the cDNA of *M. oryzae* that was cultured with 1% DMSO via PCR with primers TAS1-F and TAS1-R (fragment 4). Two kb of the downstream region of the *TAS1* gene was amplified from the genomic DNA of *M. oryzae* via PCR with primers OETAS1_DN-F and OETAS1_DN-R (fragment 5). The vector sequence of pBI121 between the right and left borders was amplified from pBI121 via PCR with primers pBI121-RB and pBI121-LB (fragment 6).

All fragments were gel purified and then cloned with an In-Fusion HD Cloning kit (Clontech) to yield pBI-OE::TAS1. *E. coli* DH5α and *A. tumefaciens* transformation was conducted in the manner described for *MGG_07803* gene disruption. *TAS1* overexpression strains were selected via PCR with primers OETAS1_CHK-F and OETAS1_CHK-R that hybridize outside of the used upstream region and the *TAS1* gene. This primer set can amplify 3.3 kb from wild-type strains and the 5.4-kb blasticidin S resistance gene expression unit containing region from *TAS1* overexpression strains (Supplementary Fig. 4).

### KS domain disruption

KS domain-disrupted strains were constructed as follows. Two kb of the upstream region of *TAS1* was amplified from the genomic DNA of *M. oryzae* via PCR with primers ΔKS_UP-F and ΔKS_UP-R (fragment 1). The hygromycin B resistance gene expression unit was amplified from pCSN45 (ref. 60) via PCR with primers 5HPH and 3HPH (fragment 2). The *TEF1* promoter region and the C-A-PCP domains of *TAS1* were amplified from pBI-OE::TAS1 via PCR with primers TEF1+CAT-F and TEF1+CAT-R (fragment 3). Two kb of the downstream region of the *TAS1* gene was amplified from the genomic DNA of *M. oryzae* via PCR with primers ΔKS_DN-F and ΔKS_DN-R (fragment 4). The vector sequence of pBI121 between the right and left borders was amplified from pBI121 via PCR with primers pBI121-RB and pBI121-LB (fragment 5).

All fragments were gel purified and cloned by using the In-Fusion cloning technology to yield pBI-TAS1-KS::HPH. *E. coli* DH5α and *A. tumefaciens* transformation was conducted in the manner described for *MGG_07803* gene disruption. The KS domain of *TAS1* disruptants were selected via PCR with primers ΔKS_CHK-F and ΔKS_CHK-R that hybridizes upstream of the KS domain and outside of the downstream region used for vector construction of *TAS1*. This primer set can amplify 4.1 kb from wild-type strains and 2.9 kb from the KS domain disruptants (Supplementary Fig. 9).

### Microarray analysis

An RNeasy Plant mini kit (Qiagen) was used to extract total RNA from static-cultured *M. oryzae* (wild type, Δ*osm1* and 1% DMSO-added condition) on day 5 of culture. Double-stranded cDNA synthesis (from 10 μg total RNA) and Cy-3 labelling and hybridization steps were performed by Roche NimbleGen (Madison, WI, USA) according to standard protocols. A custom-designed microarray platform comprising single 60-mer probes designed against 12,825 annotated genes from the genome sequence of *M. oryzae* was used^33^. Five non-overlapping 60-mer probes were designated against various parts of the single target gene, and 64,125 probes were finally designated against the total number of annotated genes. An average transcriptional intensity of five non-overlapping probes in the single gene was used as the transcriptional level of the gene.

### Metabolite extraction and analysis

*M. oryzae* culture broth (100 μl) was extracted with four volumes of ethanol, evaporated with an N_2_ stream and dissolved in 200 μl methanol to analyse metabolites. UPLC/MS analysis was performed with an Acquity UPLC H-Class system (Waters Alliance, Milford, MA, USA) equipped with a mass spectrometer (API 3200, Applied Biosystems). The UPLC conditions were as follows: column, XTerraMSC_18_ (Waters), 5 μm (2.1 × 150 mm); flow rate, 0.6 ml min^−1^; solvent A, water-containing 0.05% formic acid; solvent B, acetonitrile. After the extracted sample was injected into a column equilibrated with 5% solvent B, the column was developed with a linear gradient from 5 to 100% solvent B over the course of 3.5 min and kept at 100% solvent B for another 2 min. Mass spectra were collected in the electrospray ionization-positive and electrospray ionization-negative modes.

### Protein expression and purification from *S. cerevisiae*

*S. cerevisiae* protease-deficient strain BJ5464 was obtained from the American Type Culture Collection (ATCC 208288) and used as the host cell for TAS1 protein expression. A PCR fragment of 4,809 bp containing the *TAS1* ORF was amplified from the cDNA of *M. oryzae* that was cultured with 1% DMSO via PCR with primers TAS1-F and TAS1-R, and the amplified fragment was inserted into the YEp352 ADH1 vector^61^ that amplified with primers YEp352-F and YEp352-R via infusion reaction to construct the N-terminal-8 × His-tagged TAS1 expression plasmid YEp352-TAS1. The nucleotide sequence of the inserted fragment was confirmed. To express TAS1 as an active form, we also amplified the *PPTase* coding sequence (*MGG_17878*, 978 bp) of *M. oryzae* from *M. oryzae* cDNA via PCR with primers MoPPT-F and MoPPT-R and inserted it into YEp352 to construct YEp352-MoPPT. The *ADH1* promoter and terminator containing the *PPTase* coding sequence from YEp352-MoPPT was amplified with primers ADH1-F and ADH1-R, subcloned into the pESC-Trp-modified vector (Stratagene, La Jolla, CA, USA), which was deleted at the 1,960- to 3,302-bp region containing *GAL1* and *GAL10* yeast promoters and terminators by amplification with primers pESC-TRP-F and pESC-TRP-R to obtain pESC-Trp-ADH1-MoPPT for the expression of non-tagged PPTase.

The constructed YEp352-TAS1 and pESC-Trp-ADH1-MoPPT were co-transformed to *S. cerevisiae* strain BJ5464 with the lithium acetate/single-stranded carrier DNA/Polyethylene glycol method^62^, and transformants were selected on a synthetic glucose induction (SGI) plate (l g l^−1^ casamino acids, 7 g l^−1^ yeast nitrogen base, 20 g l^−1^ glucose and 2% agar). Transformed cells were pre-cultured at 30 °C in SGI liquid media for 24 h and then inoculated into 2 l YPD media (2% glucose, 2% bactopeptone and 1% yeast extract) with an initial optical density at 600 nm of 0.015 and cultured for 48 h at 28 °C. The cultured cells were harvested via centrifugation (5,000*g*) for 15 min at 4 °C, and then the collected cells were suspended in 5 ml cold distilled water. Suspended cells were granulized via dripping into liquid N_2_ and then homogenized with a Waring mixer with liquid N_2_. Fifty grams of homogenized cells were suspended in 100 ml protein extraction buffer (10 mM Tris-HCl (pH 8.0), 1 mM dithiothreitol (DTT), 0.1% TritonX-100 and 15% glycerol). The cell suspension was heavily vortexed for 1 min and sonicated with a Tomy UD-200 sonicator twice on ice for 30 s each with 30-s intervals. Cellular debris was removed via centrifugation (8,000g) for 30 min (Beckman GS-15). The supernatant was diluted in the same volume of 2 × bind buffer (1 mM DTT, 800 mM NaCl, 10 mM imidazole and 15% glycerol), applied to a Ni-nitrilotriacetic acid beads column (2 × 8 cm; Qiagen) equilibrated with equilibration buffer (10 mM Tris-HCl (pH 8.0), 1 mM DTT, 400 mM NaCl and 15% glycerol), and washed with 100 ml wash buffer (10 mM Tris-HCl (pH 8.0), 1 mM DTT, 50 mM NaCl, 5 mM MgCl_2_, 0.1 mM ethylenediaminetetraacetic acid and 15% glycerol) containing 10 mM imidazole. The column was further washed with 75 ml wash buffer containing 30 mM imidazole.

The His-tag-binding protein was eluted with 60 ml elution buffer (10 mM Tris-HCl (pH 8.0), 1 mM DTT, 50 mM NaCl, 5 mM MgCl_2_, 0.1 mM ethylenediaminetetraacetic acid and 25% glycerol) containing 200 mM imidazole. The eluted protein was concentrated with an Amicon Ultra-100k (Millipore, Bedford, MA, USA), and then buffer exchange was conducted with buffer A (10 mM Tris-HCl (pH 7.5) and 25% glycerol) using a PD Midi Trap G-25 column (GE Healthcare, Uppsala, Sweden) and applied to a Superdex 200 column (GE Healthcare) for size exclusion. The column was previously equilibrated with running buffer (50 mM Tris-HCl (pH 7.5), 500 mM NaCl and 10% glycerol) at a flow rate of 0.8 ml min^−1^. Eluted 8 × His-tagged TAS1 was concentrated with the Amicon Ultra-100k (Millipore), and buffer exchange was conducted with buffer A and the PD Midi Trap G-25 column (GE Healthcare). The highly purified His-tagged TAS1 protein was stored at −80 °C and used for *in vitro* enzyme assay.

### Protein expression and purification from *E. coli*

PPTase (MGG_17878) of *M. oryzae* was overexpressed with the *E. coli* BL21(DE3)pLysS strain (Takara). The *PPTase* ORF (978 bp) of *M. oryzae* was amplified from *M. oryzae* cDNA via PCR with primers pET-MoPPT-F and pET-MoPPT-R. The pET19b(+) vector sequence was amplified from pET-H6SDH^63^ with primers pET-F and pET-R, and then two fragments were infused to obtain pET-MoPPT for the expression of N-terminally 6x His-tagged PPTase. The constructed pET-MoPPT sequence was confirmed after transformation into *E. coli* BL21(DE3)pLysS. After overnight culture at 37 °C in LB with Km (50 μg ml^−1^), 2 ml pre-cultured transformed cells was inoculated into 100 ml fresh LB with Km (50 μg ml^−1^) and cultured at 37 °C for 2 h. Isopropyl-β-D-thiogalactopyranoside was added to a final concentration of 1 mM. After growth for 5 h at 30 °C, the cells were harvested via centrifugation (5,000*g*) for 15 min at 4 °C. The collected cells were suspended in 10 ml protein extraction buffer and sonicated five times on ice for 30 s each with 30-s intervals. Cellular debris was removed via centrifugation (8,000*g* for 30 min). The supernatant was purified on a Ni-nitrilotriacetic acid beads column as described for protein expression and purification from *S. cerevisiae*. Purified PPTase (Supplementary Fig. 10b) was used for the KS domain activity assay.

### Protein expression using a wheat germ cell-free system

PCR fragments containing the C–A–PCP domain (1–3,360 nt) or KS domain (3,361–4,809 nt) of *TAS1* were amplified from the cDNA of *M. oryzae* that was cultured with 1% DMSO via PCR with primers pEU_CAT-F and pEU_CAT-R for the C–A–PCP domain and pEU_KS-F and pEU_KS-R for the KS domain. The amplified fragments were inserted into the pEU01 vector (Cell-Free Science, Matsuyama, Japan) amplified with primers pEU01-F and pEU01-R using the In-Fusion HD cloning system (Clontech). The resulting expression plasmids were designated pEU01_C-His_CAT and pEU01_N-His_KS for the synthesis of C-terminally 6 × His-tagged C–A–PCP domains and N-terminally 6 × His-tagged KS domain of TAS1, respectively. Cell-free protein synthesis with wheat embryo extracts was performed with a WEPRO7240 Expression kit (Cell Free Science) using pEU01_C-His_CAT and pEU01_N-His_KS in a 226-μl bilayer format on 96-well flat-bottom plates (Iwaki) according to the manufacturer's instructions. The solubility of the translation product was confirmed with sodium dodecyl sulphate-polyacrylamide gel electrophoresis^64^. The reaction mixture was centrifuged at 13,000*g* for 30 min at 4 °C, and the supernatant was used directly for the TeA biosynthesis assay.

### *In vitro* enzyme assay

A TAS1 activity assay was conducted at 25 °C for 2 h in 100 μl reaction mixture containing 0.5 μM TAS1, 50 mM Tris-HCl (pH 7.5), 10 mM Tris (2-carboxyethyl) phosphine (TCEP), 1 mM ATP, 5% glycerol, 1 mM isoleucine and 1 mM acetoacetyl-CoA. Acetyl CoA (1 mM) and malonyl-CoA (1 mM) were also used instead of 1 mM acetoacetyl-CoA. To determine the kinetic parameters for isoleucine, both of ATP and acetoacetyl-CoA were remained constant at saturating concentrations (2 mM) and the concentration of isoleucine was varied from 0.1 to 3 mM. For acetoacetyl-CoA, both ATP and isoleucine were remained constant at saturating concentrations (2 mM) and the concentration of acetoacetyl-CoA was varied from 0.005 to 1 mM. For ATP, both acetoacetyl-CoA and isoleucine were remained constant at saturating concentrations (2 mM) and the concentration of ATP was varied from 0.01 to 2 mM. The kinetic parameters *K*_M(app)_ and *k*_cat_ and their standard errors were determined using non-linear regression to fit the data to the Michaelis–Menten equation using SigmaPlot (Systat software). All reactions were performed in triplicate. A KS domain activity assay was conducted in 260 μl reaction mixture. The first reaction was conducted with 0.8 μM purified MoPPT containing100 μl soluble crude proteins containing C-His-tagged C–A–PCP, N-His-tagged KS protein, or both, 80 μM CoA, 4 mM ATP, 2 mM MgCl_2_, 8 mM TCEP, 5% glycerol and translation buffer with the WEPRO7240 Expression kit (Cell Free Science) at 30 °C for 1 h. Then, 0.4 mM isoleucine and acetoacetyl-CoA were added and reacted at 25 °C for 5 h. For analysis of the cyclization capability of the KS domain, the enzyme reaction was conducted in 200 μl reaction mixture containing five times-concentrated and buffer (50 mM MES (pH 6.5))-exchanged 100 μl of C-His-tagged C–A–PCP or N-His-tagged KS soluble crude proteins synthesized with the cell-free system, 5% glycerol, 5 mM TCEP and 0.25 mM *N*-acetoacetyl-L-Ile-SNAC at 25 °C for 2 h. For analysis of the condensation capability of the TAS1, the enzyme reaction was conducted in the same manner as cyclization capability analysis, but using 0.25 mM L-Ile-SNAC and acetoacetyl-CoA instead of *N*-acetoacetyl-L-Ile-SNAC.

After the enzyme reaction, the reaction mixtures were extracted with four volumes of ethanol, evaporated with an N_2_ stream, and finally dissolved in 200 μl methanol for UPLC/MS analysis of TeA production. After injection of the extracted sample into a column equilibrated with 5% solvent B, the column was developed with a linear gradient from 5 to 100% solvent B over 3.0 min and kept at 100% solvent B for another 2 min. Mass spectra were collected in the electrospray ionization-positive and electrospray ionization-negative modes.

## Additional information

**How to cite this article:** Yun, C.-S. *et al.* Biosynthesis of the mycotoxin tenuazonic acid by a fungal NRPS–PKS hybrid enzyme. *Nat. Commun.* 6:8758 doi: 10.1038/ncomms9758 (2015).

## Supplementary Material

Supplementary Information Supplementary Figures 1-13, Supplementary Tables 1-4 and Supplementary Methods
