# Supplementary material for: Biosynthesis of the mycotoxin tenuazonic acid by a fungal NRPS–PKS hybrid enzyme
Source: Nat Commun. 2015 Oct 27;6:8758. doi: 10.1038/ncomms9758 (PMC4640141; doi:10.1038/ncomms9758)
Supplement: Supplementary Information — Supplementary Figures 1-13, Supplementary Tables 1-4 and Supplementary Methods [file ncomms9758-s1.pdf]

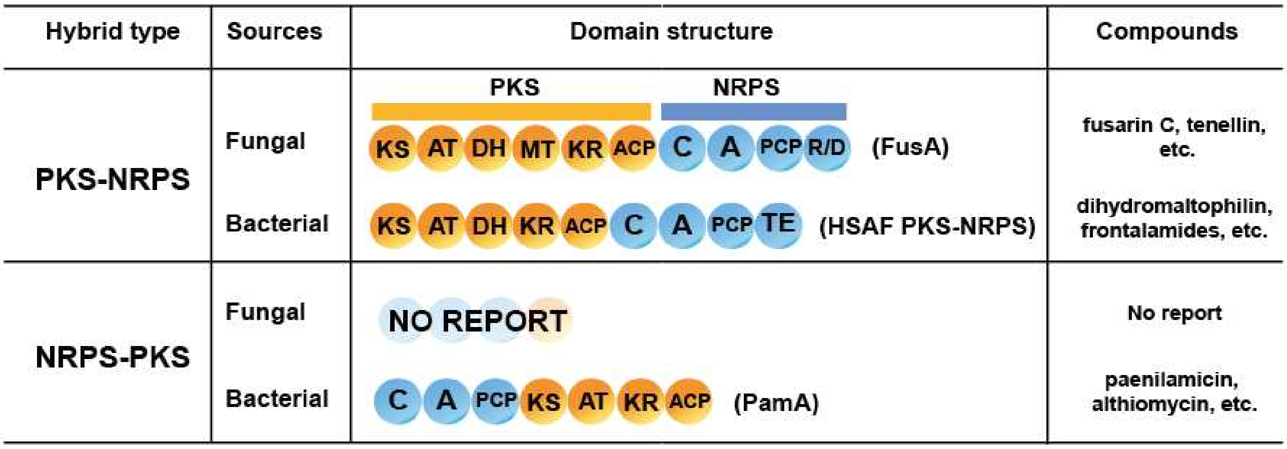

Supplementary Figure 1. Domain structure of hybrid enzymes. Domain structure of a typical fungal PKS-NRPS, bacterial PKS-NRPS, and bacterial NRPS-PKS hybrid enzyme. FusA; fusarin synthetase A from *Fusarium moniliforme*. HSAF PKS-NRPS; heat-stable antifungal factor, dihydromaltophilin synthesis core enzyme from *Lysobacter enzymogenes*. PamA; paenilamicin synthetase A from *Paenibacillus larvae*. Blue indicates the NRPS portion, yellow indicates the PKS portion.

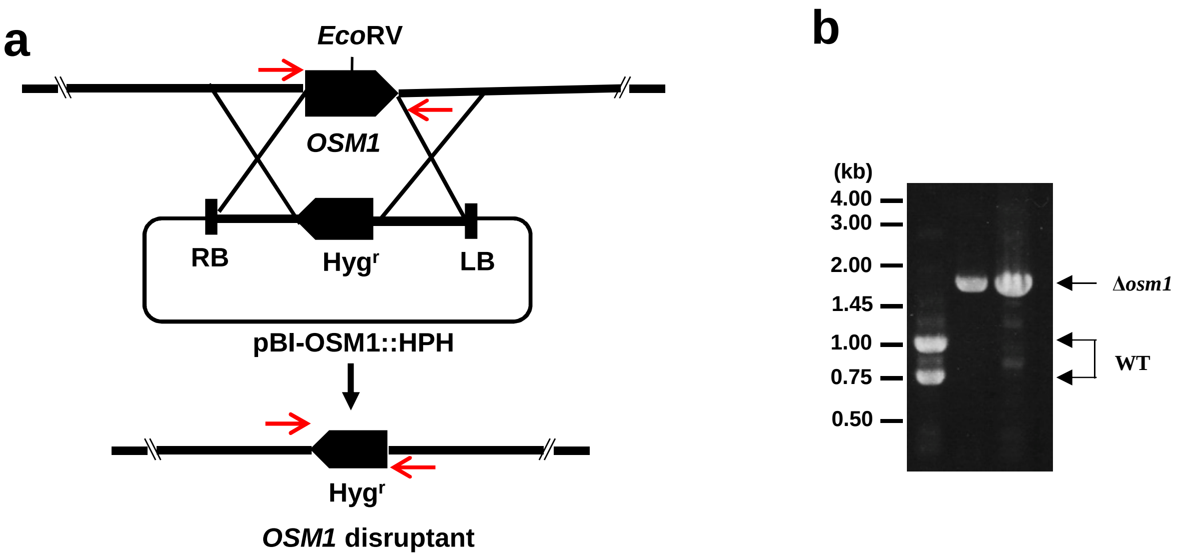

Supplementary Figure 2. Disruption of the *OSM1* gene. (a) Scheme of the *OSM1* gene disruption. Red arrows indicate the position of primers used for disruptant selection. (b) Confirmation of *OSM1*-disruption by Polymerase chain reaction (PCR). WT and *OSM1* disruptants were analysed by colony PCR followed by *Eco*RV digestion. Arrows indicate the expected band position of the PCR analysis

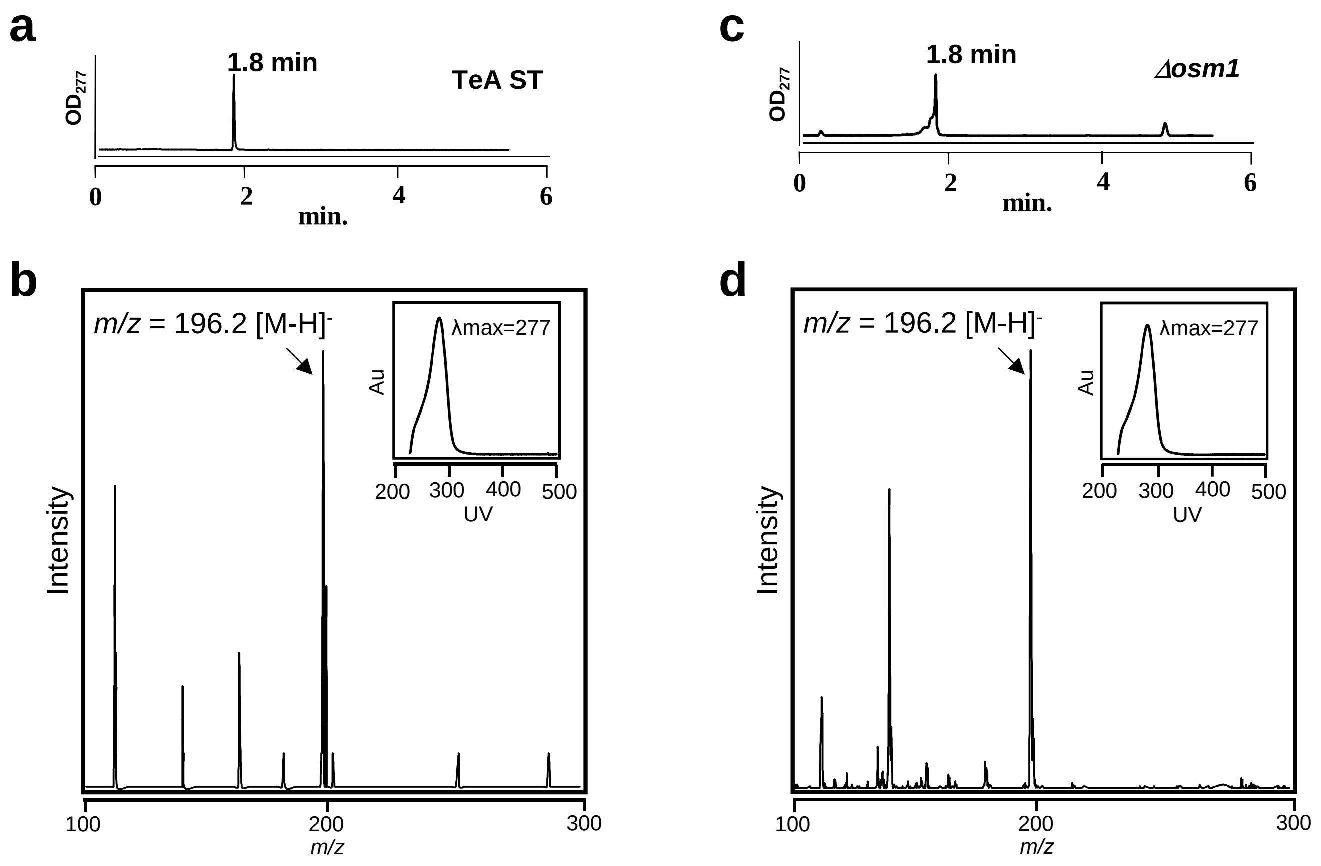

Supplementary Figure 3. Ultra performance liquid chromatography/mass spectroscopy (UPLC/MS) analysis of tenuazonic acid (TeA). (a) UPLC analysis of the TeA standard showed an ultraviolet (UV) peak at 1.8 min. (b) Mass spectra from the TeA peak (1.8 min). Small panel in the mass spectra shows the UV absorbance profile of the TeA peak detected with UPLC. (c) UPLC analysis of metabolites extracted from *Magnaporthe oryzae Δosm1* static-cultured on day 5 of culture showed the major UV peak at 1.8 min. (d) Mass spectra from the major peak (1.8 min). Small panel in the mass spectra shows the UV absorbance profile of the major peak detected with UPLC.

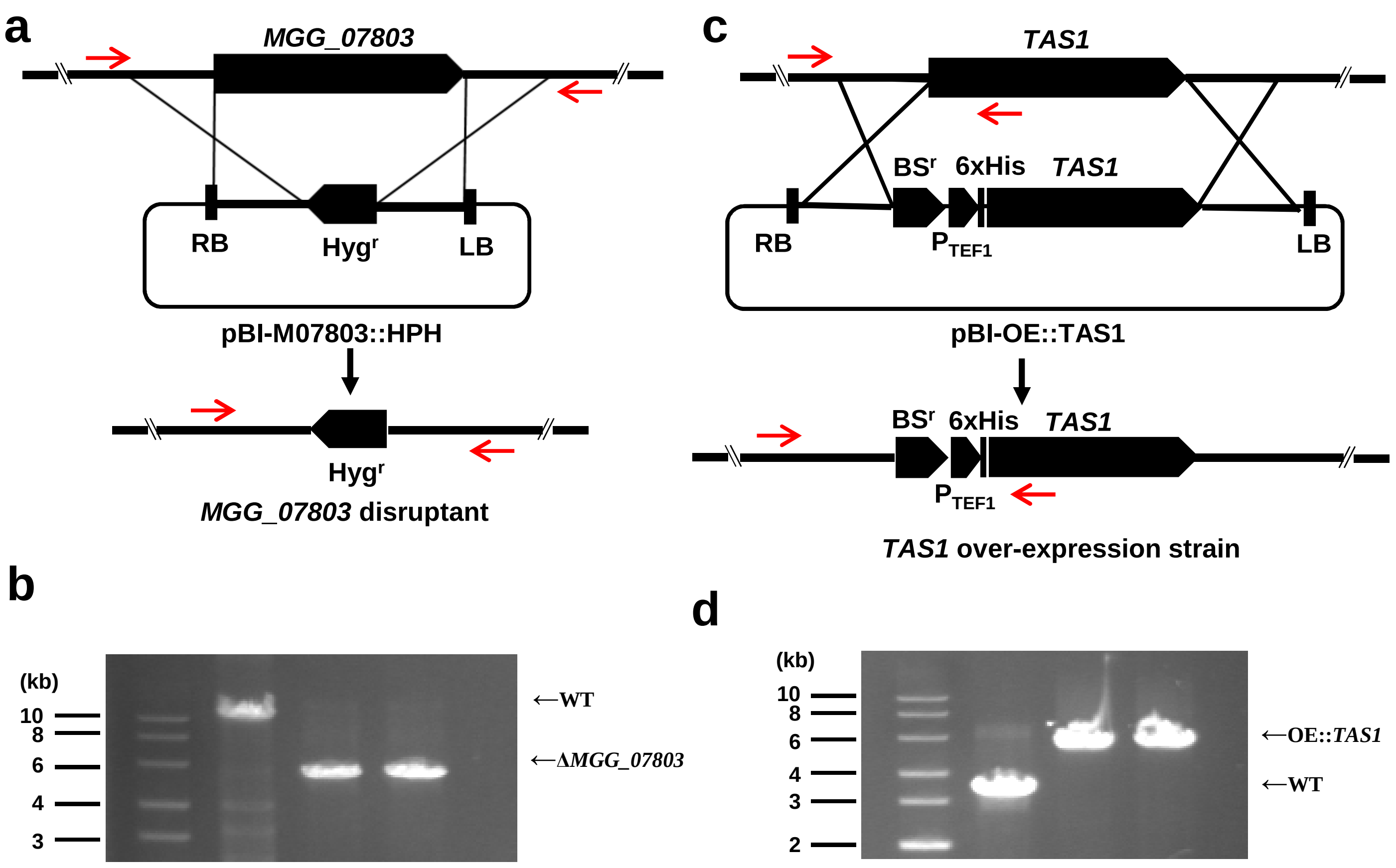

Supplementary Figure 4. Construction of the *MGG_07803* gene disruptants and the TeA synthetase gene (*TAS1*) overexpression strains. (a) Scheme of *MGG_07803* gene disruption. Red arrows indicate the position of primers used for disruptant selection. (b) PCR analysis of WT and disruptants. The WT fragment amplified from genomic DNA and the fragment of the disruptants amplified with colony PCR. Arrows indicate the expected band position of the PCR analysis. (c) Scheme of the *TAS1* overexpression strain. Red arrows indicate the position of the primers used for overexpression strain selection. (d) PCR analysis of WT and overexpression strains. The WT fragment amplified from genomic DNA and the fragments of overexpression strains were amplified with colony PCR. Arrows indicate the expected band position in the PCR analysis.

```
ATGTCTGTCAGGCCACTACCTTTTCTCACGGGGCCAGCCCCAAGCCCTGATACGGCGGCTGGCTTCAAGCCCAGCCCACCCACAGGCAAC  90
CTCGTTTCTGTTTCCCCGCTATCCAAGGCGCAGATGGCCCTGTGGTTCGACTACCTGCAACACCCAACCTCTACACACTACTTCTTGACC  180
CTCAAGGTTGAGCTGGACAAGCAGCCTCTCAGCCTGGACAAGATAATACAAGTCATCCGGGGCTTGGGCAAGCAACATGCCATGCTGCGC  270
ACAACCTTCCACGTGGACACAGATACAGATGACATGTCAAAGTCGTACATGGCCGTCCACGATGATTCGTGGGACCAGGAGATCCACGTC  360
CTGATGAACGACGCTCAGCTCTACGAAGCGCTGCGGAAGCCGTTCCAGCTGTCCTCGGAATCCCCCGTCCGCTGGGTTGTCCAGATGAAG  450
CTCCAGCCCGGCAGCGCTCGGAGCACCTACACCGTCTACGCGGCGGGGCATCACATCGGCGTGGACGGGGCGTCCATGAGCGTGCTGTCG  540
AACCAGCTCCTGGAGGCCGTGGCGTCCGAGGTTGAGGACCAGCCCGACCACAGCGGCCCCCACTACGGGGACTATATCCAAAGACAGGCA  630
AGATATCTGCGCAGTTCAGCAGGTGCGGCGGCTGGCAGGTTCTGGCTCTCGCAACTGCGACACACCCAGCCGTTCCGGTGGCGGATGGAG  720
CCGCCAGAAGAGATCAACACCCCAAACTACCGTCAGCTGGACACGTGGAACTTTTTCCCGACCGCTGAAATCCAGGAATGGGGCAACCTA  810
TACAAGACGTCCTGGTTTCGCGTGGCCACGTCCATAGTGGGGCTCGTCACTGCGGCCATGGCTGAGCCCCAAGCCCACCACGACCATGCT  900
CTGATGGTGGCGTTTGGAGCCAGGCCCAGGGGCTTTGAAAACAACGTTTCGCACATGGCCAACACCATGCCCGTCAAGTTTCCCCTGTCG  990
TCGCTGCTGCGGGACGACGCCACCTTTTCGGACGCGGTCAAAGCCATGGGCCGCAACGTTTCCACGGCCAAAAAGCACGAAAACTTTCCA  1080
TTCATGTCGCTCATGGAGCAGGCAAATCGGCACATGGACCCCACGCTGCTGGACTTCAAAGTGGCCATAACATACTCGCCCAAACTTGCC  1170
AACAAGAGCTGCGAGCTGTTTCCCGTGGAGGGCATATGGGACCTGTTCTTTTGCTTCCTCGAGCAGGAGGACGGCGTGGCCCTGGGAGTC  1260
ATCAGCAACCCGCGTGTTTTCGGCGCGGAAGCTCTCGGCCAGCTGCAAAGCTTGTTCAACGAGGTTTTTGCGCTGTCAAAGGCCCGCCCG  1350
AGCTTTAAGCTGTCCGACTTGGCGTTTCTGCAAAACAGGACGCCCGCTCGGTTCATCTCGGGCCCGGCCCTGGACGACGTCGAGAGCATC  1440
AGCAAGTCGAGGGTTTACCGGCTGATCAAGGCCAGAGCGGCGAGCCAGCCCGACCTCGTCGCTCTCATGTCGGCAGAAAAAGGTGTGCAA  1530
ATGACGTACCGCGAACTCGCAGCTCAGAGCAGCCAAGTAGCCCACTTTCTCCAAAAACAGCGTCTTTGCAAGGGCGATGCAGTCCTCGTC  1620
CACCTGGAACGCGGCTTCGCCCAGATCGTATGGATCCTCGGCGTCATGGAGGCGGGTGCCTGCTATGTAGCACTCGACAAGACCTGGCCC  1170
GCAGCCAGGAAGGAAGCCATCCTGAGGACGGCCAACGGAAAGCTGTTGGTCACGGACGACGAGCAAATGGACTTTGAGAAGCAAGACACG  1800
ACCGTCGTGTTCCTTGCCCCGTCCGCGGCCGAGATAGCCAGCATGCCCCAGAGCACGTGCGAATGCGAGGTTGCGGATGACGATCTGGCC  1890
TATGTCGTCTTCACATCGGGGTCCACAGGCCAGCCCAAGGGCGTCATGGTCGAGCACTCCAACCTGAGCCACTACGTCAGCGCGACGCGC  1980
AGCCTTGTCAAGACCGGACCCCATTCCCGCATGCTGCAGCTCGCGTCCTTTGCCTTTGACGCCATAGTGCTCGAGTACGCCGTCACCCTC  2070
GCCCACGGCGGTACCTTGTGCTTTGCCAACCACCCAGAGGTGCTGGTGGGCGAGTACCTGGCCGACGTTATCGACAGCAACCAGGTCAAC  2160
TTCTTCCACTGCACACCTTCGGTCTTGTCGACCCTTCCGGCAGGGAGGCGGCTGCCCTCGCTGCGAATCGTCTCCGTGGGAGGCGAGGCC  2250
TCGCCACCTGGCCTGCTCGACCACTGGAGGAAGAGGGTGGAACTGCTGCACGCGTACGGCCCGACCGAATGCACTGTCATCTGCACACTG  2340
GAAAGCCTGACCCAAGACGAATCGACCCAGACCGCCATCGACGCCACCGTCATCGGCAAGGCGCTGCCCAACTTGGACATTCGCATATGC  2430
GAGGAAGGCAAGCTGGAGCCTCTTGCACCCAACCAAGTCGGCGAGATTTGCGTCGTCGGACCGCAGGTCTCCCGCGGGTACATGGGCCAG  2520
GAAGAGCTCACGGCCAGCAAGTTCCACAACATCACTCTTGCCGACGGCCACCCGAGCCGGCTATATCGCACTGGCGACAAGGGTTTCATT  2610
GACGACGACGGCAAGCTGCACATTCAGGGCCGGATCGGAAACCGCGAGATCAAGGTCCGGGGATACCGACTGGACCTGTACGAGGTGGAG  2700
AAGAACGTCATGGCTTTCGACCCCGAAGTCACCCAAGTGTCGATCCAGCAAGTTGGAGAGTCCCTGGTGGCACTCGTGGTGCCGGCCTCG  2790
ATCGACTGTGACCGCATCCGCAGCAAACTGCTCAAGGACATGCCCCGTTATGCAGTGCCCACTCGCTTCATCCGAGTCGCGAGCCTGCCG  2880
CTGAACACGAACGGAAAAATCGATCACACCCAGGCCAGCAGTCTTGCGGCAGAGCTGGTGATGCATGATACGGTCTTGCCCACGGTCGAT  2970
GCCACTCCTACCCCTACTGCTGCTGTACGAGCTGTCGGCGTGACGGAAGAGAATCTGCGTCTCAAGACAAAAGAAAATGGCATGGAAAGA  3060
CAGGAGATGCTTCGCAGGCACCTCACAGCCGAGGTCACAGCCTTGTGGGCCAAACTTCTGGGTTCTTCTCGGCAGTTCGACCCCGAAGTC  3150
GGATTCTTCGATGCTGGCGGCCACAGCCTCTTGTTGACCCAGCTCCACAAGCTGATCAAGGAGCGTTTCGGCACGGGGTCGCGACCCAGC  3240
CTCCTGGACATCTTTAGCATGAGCTCGATCCGGAAACAGGTCGACTGCCTCATGGGGATTGTGGACCAGGATGCCATGCTCGGATCCGAG  3330
CCCACTGGTGGCAGCAGCTCTCGGTCGCAATCCAGACGGTCGGCAGAAACCAGCTCTTCTTCCACCAGCGCTCCAAGCAGCGTTCCTGTC  3420
GACGCCGAAAGGAACCTGTACGCCATCGTGGGCATCTCGTGCCGGTTCCCCGGCGCCAATACCGCCGAGCAGCTGTGGAACGTGCTCATG  3510
GAGCAGCGCGATGCCATCACCACTTTTTGCCCCGCCGAAAACCTCGGCTTCGCCCTCGAGGAGAACAGCGTGTTCGTGCCGCGCTACGGC  3600
ATGATCGACGCGCTCAAGGACTTCGAGCCGTCGGCGTACTCCATGTCCGACGCCGAGGCCCAGACCATCGACCCCCAGAAGCGCGTCTTC  3690
CTGGACGTGGCCGCCGACGCCCTGGCCGACGCGGGCACCTCGGCCAGCCCCGGAAACCCGCTGGACCCCGTGGGCGTGTTCGTCGGCGCG  3780
GCGACCAACACCTTCCTCTCCTCGCGGGACAACCCGGGGAGCAAGCCGCCCGGCGACGAGGAGCCGCAGTCCTTTGCCAACCACTACCAG  3870
CAGCTGCTCGACTGCCCCATCGGCACCTTTGCCTCGTTCAAGCTCAACCTGACCGGGCCGGTGGTGACGCTCAACACGGCGTGCTCCTCG  3960
GCGCTCGCCGCGCTGCACCTGGCCTGCGCCTCGCTCTCGCACGGCGACTGCAACGCCGCCGTGGTGGGCGGCGTGTCCATGGCCTACCCG  4050
CAGGAGGGCGGCTACGTGACCGCCCGGCCGGGCGGCGACTCGTCGGCCGTCTTTTCCCCCTCGGGCGTCTGCCACCCGCTCGACTCGCGC  4140
GCCGACGGCTGCGTGCCGGCCGACGGCGCGGCCGCGCTGGTGATCAAGCGGCTGGCCGACGCCCGCGCCGATGGCTGCCGGGTGTACGCG  4230
GTGATCGAGGGCGTGGCAGTCAGTGCCGACGGGTCCGACGACAAGGCCGGGCTGGGCGTGCCCAGCTCCTCGGGCCAGAGCCGCACCGTC  4320
GAGGCGGCGCTACGCAGGGCGGGGCCCCAGGCGCTCAGCCGCCTGCGCTACGTCGAGATGCACGGCTCCGGCACCCCCTGGGGCGACGCG  4410
CTCGAGGTGCAAGGGCTCAAGATGGCATTCGACCGCCTCAGCAAGACCGGTGCCGCAGAGCAGTCCGGCACGGGCCGGGCCCAGCCGGAA  4500
GCGGACAGGATCTACCTGGGCTCCAACAAGGGCAACTGCGGCAACACCGAGGCCGCCTCGGGACTGCTGAGCCTGATCAAGGCGTCCATG  4590
GCCCTCAACCTGGGCGTCGTGCCTCCGCTGCCGAACCTGGCCGAGCCCAACCCCAAGTGCGAGTTCGAAGAGACCAAGTTCGAGCCGCTC  4680
GGGAAGCAGCTGGCCCTCGCGCCGGGGGACCGAGTAGGTGTCACCAGTCTGGGTTATGGCGGCAGCAATGCGCACGTGGTCTTGGCCTCG  4770
GCGCAGCTTTTCGGGGTGGAACAGAAGGCTTTCTTTTGA                                                    4809
```

Supplementary Figure 5. ***TAS1*** **open reading frame determined with rapid amplification of complementary DNA ends-PCR.** The red letter indicates the condensation (C) domain, the blue letter indicates the adenylation (A) domain, the green letter indicates the peptidyl carrier protein (PCP) domain, and the purple letters indicate the ketosynthase (KS) domain. Each domain was determined with Pfam (http://pfam.xfam.org/search).

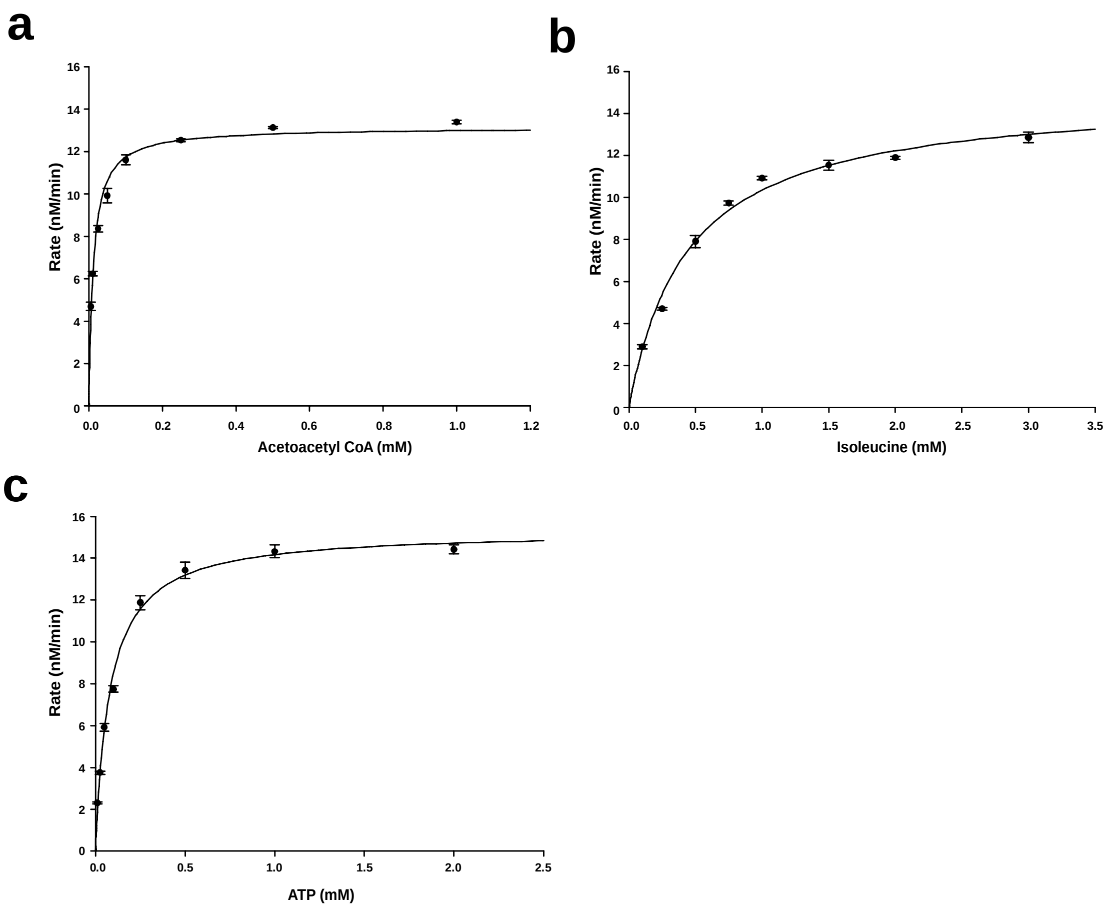

Supplementary Figure 6. Determination of kinetic parameters of TAS1. Data were fitted to the Michaelis-Menten equation using nonlinear least squares regression. (a) Initial velocities as a function of acetoacetyl CoA concentration were measured. The concentration of isoleucine and ATP were kept at 2 mM. (b) Initial velocities as a function of isoleucine concentration were measured. The concentration of acetoacetyl CoA and ATP were kept at 2 mM. (c) Initial velocities as a function of ATP concentration were measured. The concentration of isoleucine and acetoacetyl CoA were kept at 2 mM.

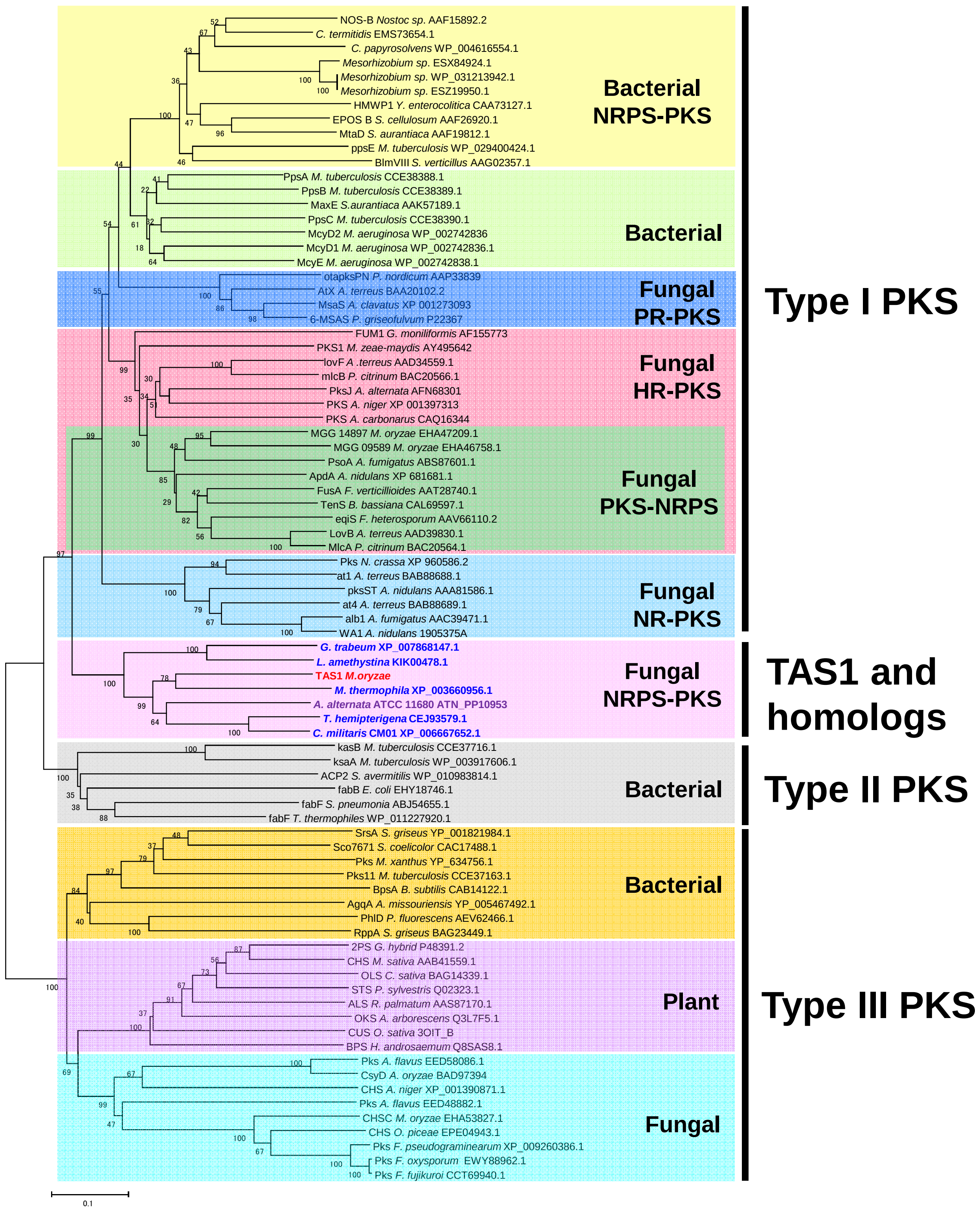

Supplementary Figure 7. Phylogenetic analysis of the TAS1 KS domain. Phylogenetic analysis of the KS domain in TAS1 and other KS domains from types I, II, III PKSs, fungal PKS-NRPSs, and the top five homologs from fungal and bacterial sources extracted with a TAS1 homolog search. Domains were aligned with MUSCLE, and the tree was constructed with the neighbour-joining method. Scale bar, 0.1 substitutions per site. The KS domain in TAS1 is shown in red. The KS domains of the TAS1 homologs in *Alternaria* sp. in purple and other KS domains of the TAS1 homologs are shown in blue. KS domains were extracted using a PKS-NRPS analysis website (http://nrps.igs.umaryland.edu/nrps/) or Pfam (http://pfam.xfam.org/search). GenBank accession number of each protein is included.

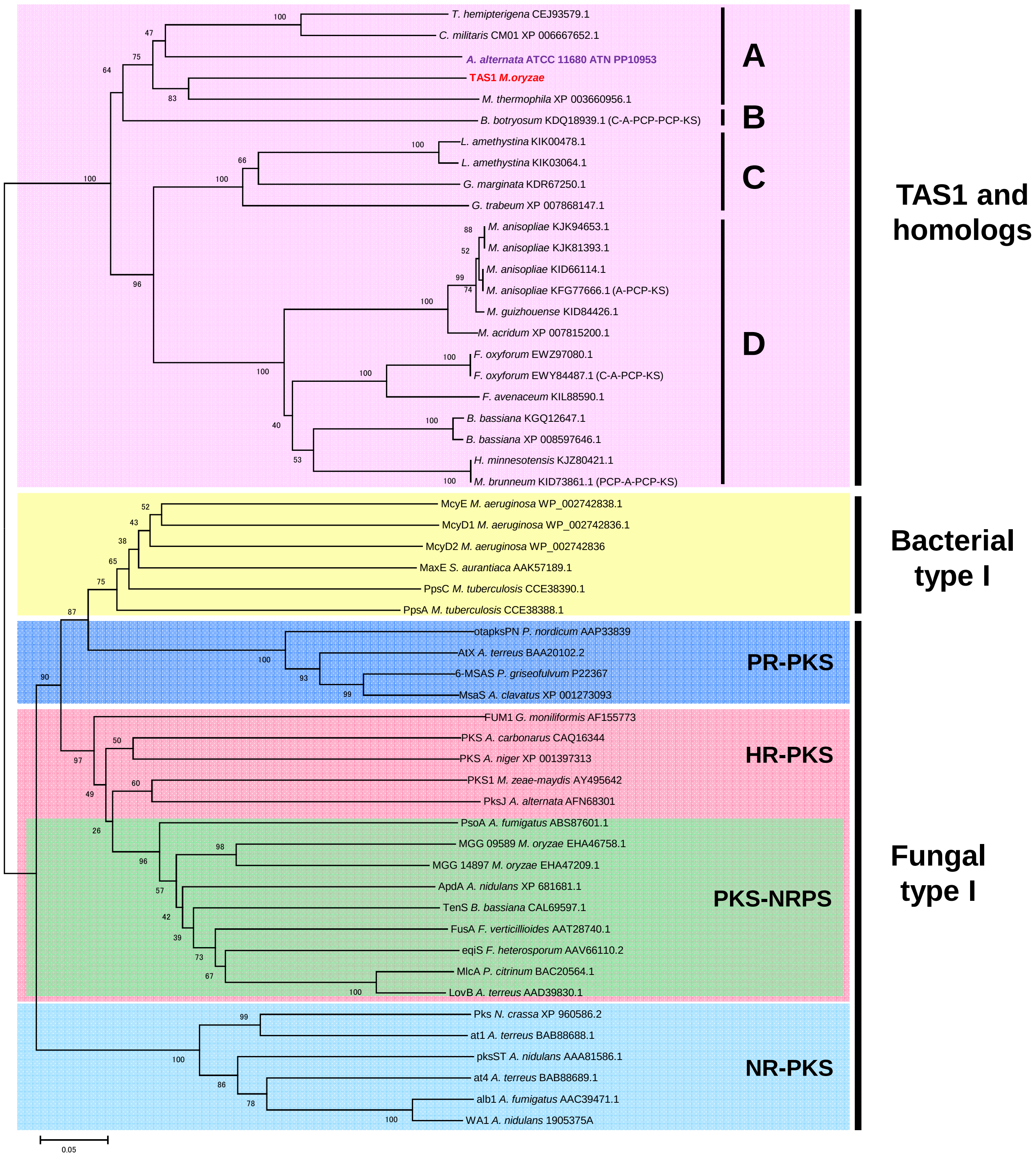

Supplementary Figure 8. Phylogenetic analysis of the TAS1 homolog KS domain. Phylogenetic analysis of the KS domain in TAS1, other KS domains from TAS1 homologs, types I PKSs, and fungal PKS-NRPSs. Domains were aligned using MUSCLE, and the tree was constructed using the neighbour-joining method. Scale bar, 0.05 substitutions per site. The KS domain in TAS1 and the *A. alternata* homolog is shown in red and purple, respectively. A, B, C, D indicate sub-clades of the TAS1 homologs. Domain structures of TAS1 homologs which were have a different domain structure than TAS1 were showed. The KS domains were extracted using a PKS-NRPS analysis website (http://nrps.igs.umaryland.edu/nrps/) or Pfam (http://pfam.xfam.org/search). The GenBank accession number of each protein is included.

a

b

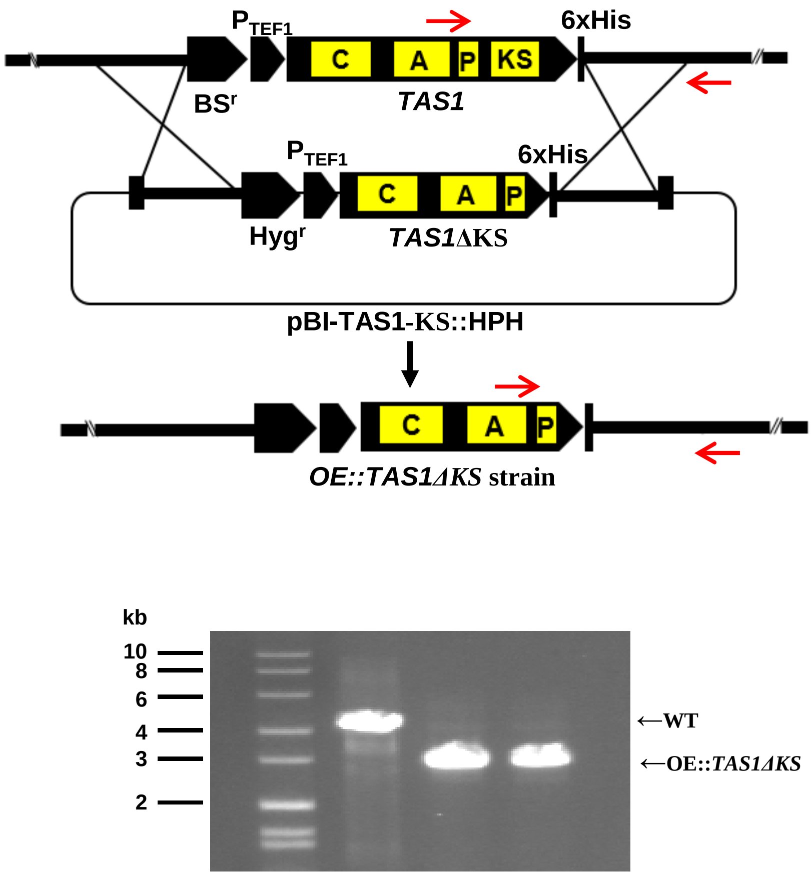

Supplementary Figure 9. Construction of KS domain disrupted TAS1 over expression strains. (a) Scheme of KS domain disrupted TAS1 over expression strains. Red arrows indicate the position of primers used for over expression strain selection. C: condensation domain, A: adenylation domain, P: PCP for peptidyl carrier protein domain. (b) PCR analysis of WT and KS domain disrupted TAS1over expression strains. WT fragment amplified from genomic DNA, fragment of KS domain disrupted TAS1 over expression strains amplified by colony PCR. The arrows indicate the expected band position of PCR analysis.

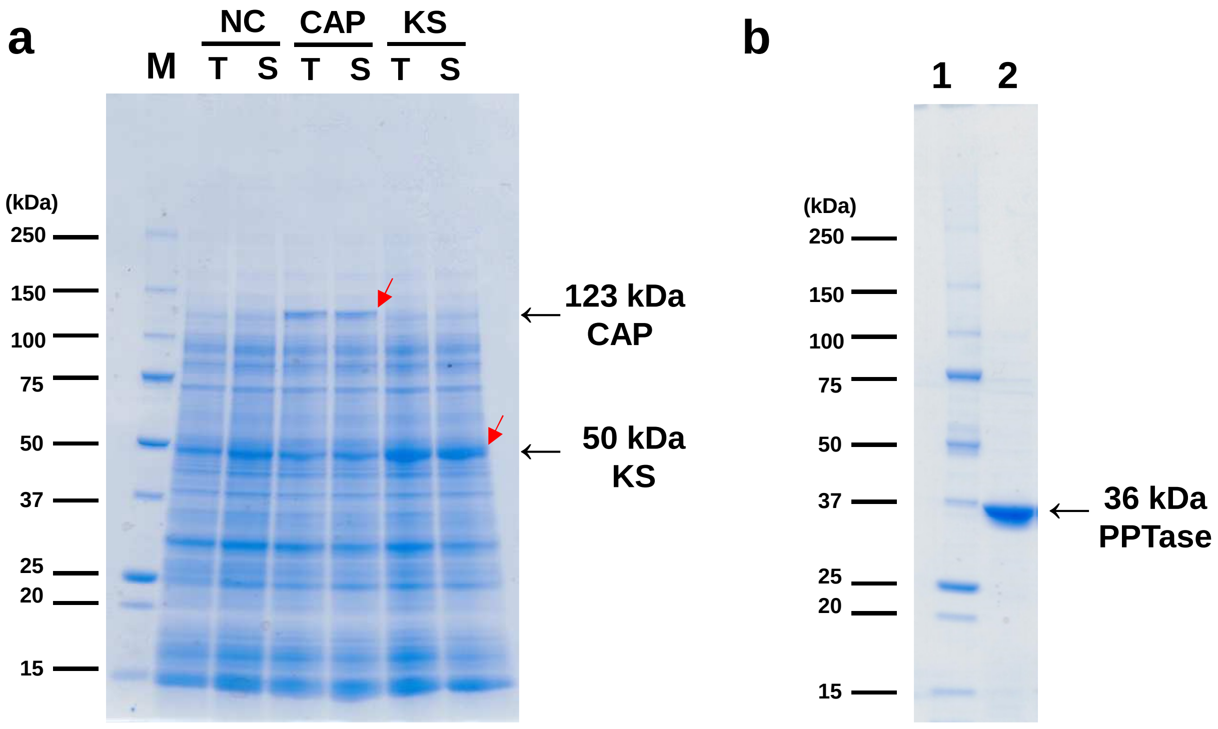

Supplementary Figure 10. Sodium dodecyl sulfate-polyacrylamide gel electrophoresis (SDS-PAGE) of proteins synthesized with a cell-free system and purified 4′-phosphopantetheinyl transferase (PPTase). (a) Proteins synthesized with the cell-free system were analysed with a 5% to 20% SDS-PAGE gradient gel. M, molecular mass markers; T, total synthesized protein; S, soluble fraction of synthesized protein. NC, synthesized proteins without vector; CAP, synthesized proteins using condensation, adenylation, and peptidyl carrier protein (C-A-PCP) domain-expressing vector pEU01_C-His_CAT; KS, synthesized proteins using KS domain-expressing vector pEU01_N-His_KS (5 µl each from a 226-µl protein synthesis were loaded). Red arrows indicate the synthesized soluble C-A-PCP and KS domain proteins. Black arrows indicate the expected size positions of the C-A-PCP and KS domain proteins. (b) Purified C-terminally 6×His tagged PPTase protein was loaded and analysed with a 5% to 20% SDS-PAGE gradient gel. Line 1, molecular mass markers. Line2, purified PPTase (5 µg).

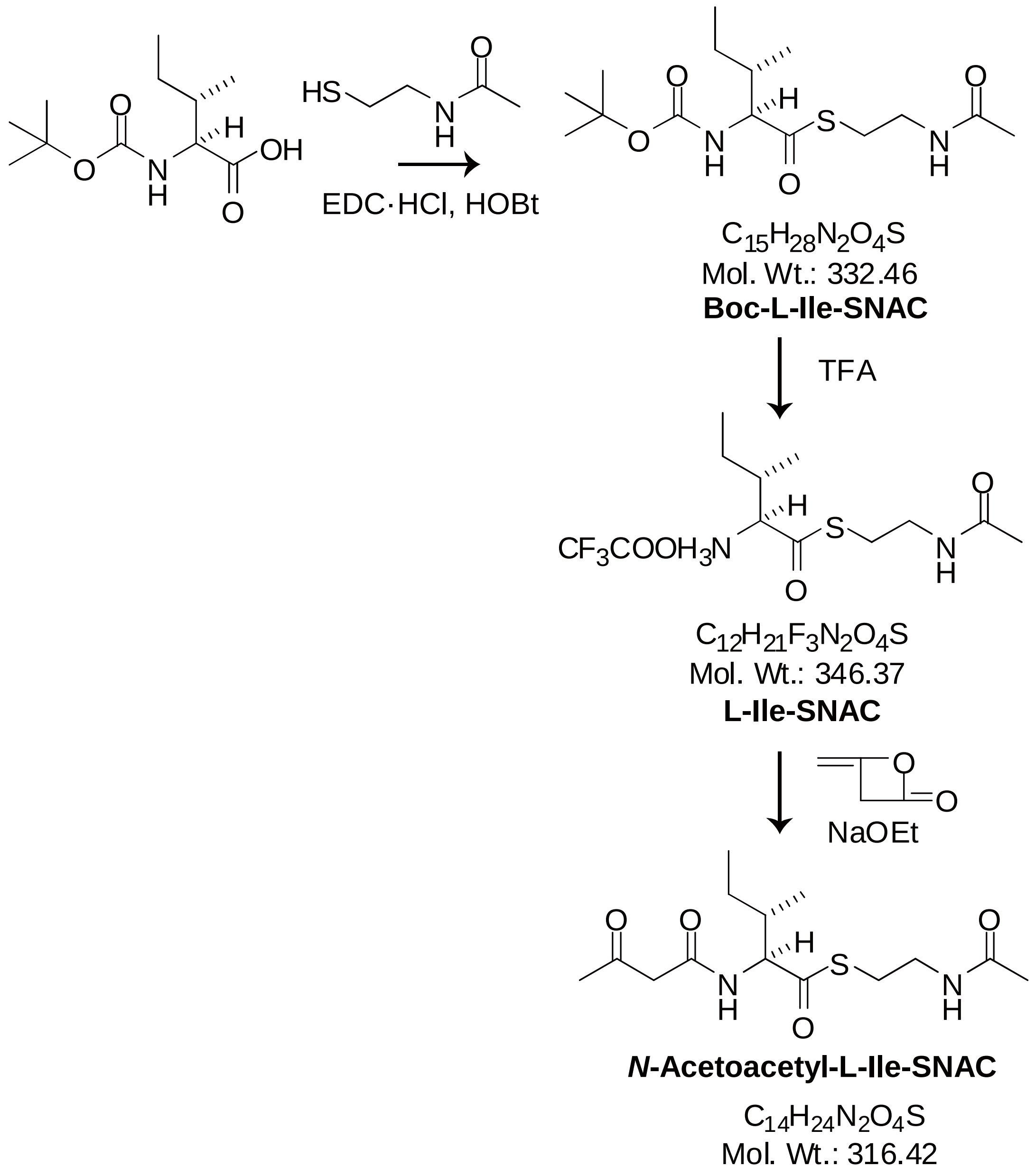

Supplementary Figure 11. Synthetic flow chart of artificial intermediate (*N*-acetoacetyl-L-Ile-SNAC). Synthetic details are described in the Supplementary methods section (see Artificial Intermediate Synthesis). SNAC, *N*-acetylcysteamine.

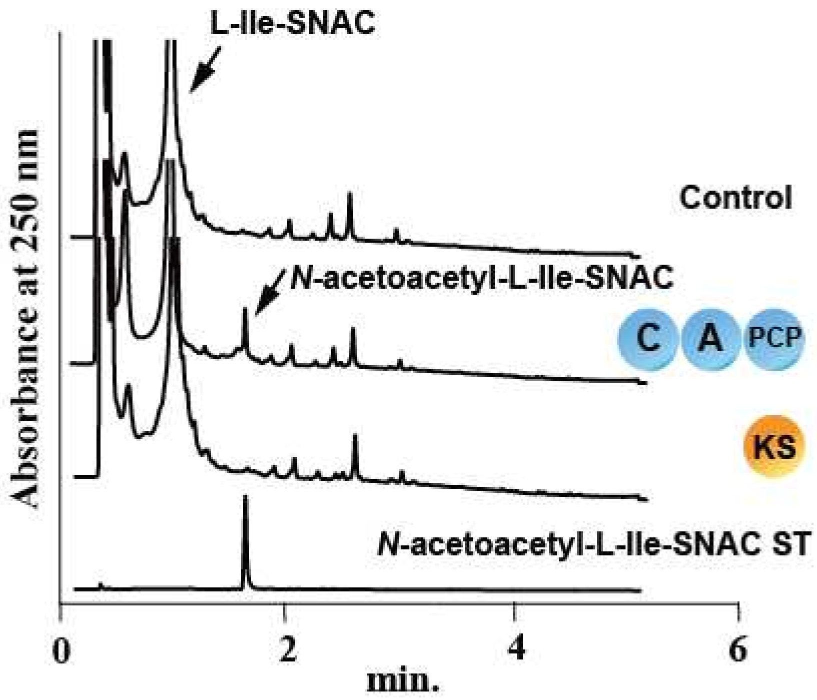

Supplementary Figure 12. Condensation capability of TAS1. UPLC analysis of enzyme reaction products. Soluble C-A-PCP domain proteins and KS domain proteins were used to investigate condensation capability between acetoacetyl-CoA and an artificial intermediate L-Ile-SNAC. A control reaction was conducted with cell-free synthesized soluble protein without messenger RNA. *In vitro* reaction conditions are described in the methods section. ST; standard compound.

```
         PpsC (Bacterial PKS type I)            IDTACSSSLVAVHLACQSLRGRESDMALVGGTNLLLSPGPSIACSRWG-----MLSPEGR
         EPOS B (Bacterial NRPS/PKS type I)     VQTACSTSLVAVHLACMSLLDRECDMALAGGITVRIPHRAGYVYAEGG-----IFSPDGH
Type I   lovF (Fungal HR-PKS type I)            IDTACSTTLTALHLAIQSLRAGESDMAIVAGANLLLNPDVFTTMSNLG-----FLSSDGI
         alb1 (Fungal NR-PKS type I)            VDTACSSSLAAIHLACNAIWRNDCDTAISGGVNLLTNPDNHAGLDRGH-----FLSRTGN
         FusA (Fungal PKS/NRPS type I)          IDTACSSSLVAVYDAVTALRNGVSKIACAGGANLILGPEMMISESKLH-----MLSPTGR
         TAS1                                   LNTACSSALAALHLACASLSHGDCNAAVVGGVSMAYPQEGGYVTARPGGDSSAVFSPSGV
         CHS (Plant PKS type III)               YQQGCFAGGTVLRLAKDLAE----NNKGARVLVVCS-EVTAVTFRGPSDTHLDS---LVG
         OKS (Plant PKS type III)               YMQGCYAGGTVMRYAKDLAE----NNRGARVLVVCA-ELTIIGLRGPNESHLDN---AIG
Type III CHS (Fungal PKS type III)              SGVGCAGGCAALRVASTLAAAATYRKQEARILVVAC-ELCSIHLRGELHAASLAEMTTIA
         CHS (Fungal PKS type III)              HGIGCSGGLAALRTAANLALGHAMRGKPARILVVTL-EVSTMMVRSELDSINESQDTRIG
         AggA (Bacterial PKS type III)          GHMGCYAALPGLGTVSDFVVA---RGRPA--LLLCA-ELTSLHLQPAGTRTDIQQ--IVS
         SrsA (Bacterial PKS type III)          FGLGCVAGAAGTARLHDYLL-----GRPDDVAVLLSVELCSLTFQ----RHDASPANLVA

         PpsC (Bacterial PKS type I)            CKTFDASADGYVRGEGAAVVVLKRLDDAVRDGNRILAVVRGSAVNQDG--ASSGVTVPNG
         EPOS B (Bacterial NRPS/PKS type I)     CRAFDAKANGTIMGNGCGVVLLKPLDRALSDGDPVRAVILGSATNNDG-ARKIGFTAPSE
         lovF (Fungal HR-PKS type I)            SYSFDSRADGYGRGEGVAAIVLKTLPDAVRDGDPIRLIVRETAINQDG--RTPAISTPSG
         alb1 (Fungal NR-PKS type I)            CNTFDDGADGYCRADGVGTIVLKRLEDAEADNDPILGVINAAYTNHSA--EAVSITRPHV
         FusA (Fungal PKS/NRPS type I)          SRMWDASANGYARGEGVAAIMMKTLSQALADGDHIQGVIREIGVNSDG--RTNGITLPSP
         TAS1                                   CHPLDSRADGCVPADGAAALVIKRLADARADGCRVYAVIEGVAVSADGSDDKAGLGVPSS
         CHS (Plant PKS type III)               QALFGDGAAALIV--GSDPVPEIEKPIFEMVWTAQTIAPDSEGAI-DGHLREAGLTFHLL
         OKS (Plant PKS type III)               NS-LFGDGAAALIV--GSDPIIGVEKPMFEIVCAKQTVIPNSEDVI-HLHMREAGLMFYM
         CHS (Fungal PKS type III)              PA-LFSDGASAFVL-CNPLGMSDKTPKQFAVVDQRTGVTPGTLDEM-SYKVTTHGFLATI
         CHS (Fungal PKS type III)              VA-LFSDCGSAVVL---SNGIGPAAEPIYDLLGWDHRIIPDTEEDL-GFDVDPVGWKVVL
         AggA (Bacterial PKS type III)          HA-LFSDAAAACVVTPGGFGYGVSEVAAVTDATTADHM---------TWEVTDAGFRMGL
         SrsA (Bacterial PKS type III)          TA-LFGDGAAALV----ALG-GRRAVSGPEIVATRSRMYPDTEHVM-GWDVGSTGFRVVL

         PpsC (Bacterial PKS type I)            PAQ-----------QALLAKALTSSKLT--AADIDYVEAHGTGTPLGDPIELDSLSKVF-
         EPOS B (Bacterial NRPS/PKS type I)     VGQ-----------AQAIMEALALAGVE--ARSIQYIETHGTGTLLGDAIETAALRRVFG
         lovF (Fungal HR-PKS type I)            EAQ-----------ECLIQDCYQKAQLD--PKQTSYVEAHGTGTRAGDPLELAVISAAF-
         alb1 (Fungal NR-PKS type I)            GAQ-----------AFIFNKLLNDTNTN--PHEIGYVEMHGTGTQAGDAVEMQSVLDVFA
         FusA (Fungal PKS/NRPS type I)          EAQ-----------KFLIRQTYKKAGLDVFKDRCQFFEAHGTGTPAGDPLEARAIHEAF-
         TAS1                                   SGQ-----------SRTVEAALRRAGPQA-LSRLRYVEMHGSGTPWGDALEVQGLKMAFD
         CHS (Plant PKS type III)               KDVPGIVSKNIDKALVEAFQPLGIS-----DYNSIFWIAHPGGPAI-LDQVEQKLALKPE
         OKS (Plant PKS type III)               SKDSPETISNNVEACLVDVFKSVGMTPPE-DWNSLFWIPHPGGRAI-LDQVEAKLKLRPE
         CHS (Fungal PKS type III)              SKSIPKLAVASIQAPFQSLIQSNGMS--SASPTDFHWALHPGGRAV-IQGAQDALNLPDD
         CHS (Fungal PKS type III)              SPRVPKMTASQLQPTFADLMASVKLPPSYQAAADFDWAMHPGGATI-LSGAERVLDITPH
         AggA (Bacterial PKS type III)          SAQVPSVLSAHVRDLVDDVLAKRGLRIADIDG----WAVHPGGPKI-LDVVQERLELDDA
         SrsA (Bacterial PKS type III)          DPAVPDVVRQYLADDVREFLDEHGLKPKDVAH----WVCHPGGPKV-LEAVTEVLDLPDG

         PpsC (Bacterial PKS type I)            --SDRAG------------SDQLVIGSVKTNLGHLEAAAGVAGLMKAVLAVHNGYIPRHL
         EPOS B (Bacterial NRPS/PKS type I)     RDASARR------------SCA--IGSVKTGIGHLESAAGIAGLIKTVLALEHRQLPPSL
         lovF (Fungal HR-PKS type I)            ------P------------GQQIQVGSVKANIGHTEAVSGLASLIKVALAVEKGVIPPNA
         alb1 (Fungal NR-PKS type I)            PDYRRGP------------ANSLYLGSAKSNIGHGESASGVTSLVKVLLMLKQNMIPPHC
         FusA (Fungal PKS/NRPS type I)          FTDGDIV------------SEPMYVGSVKTAIGHLEGCAGLAGLIKALEAVKRGVIPPNQ
         TAS1                                   RLSKTGAAEQSGTGRAQPEADRIYLGSNKGNCGNTEAASGLLSLIKASMALNLGVVPPL-
         CHS (Plant PKS type III)               KMR--------------------ATREVLSEYGNMSSACVLFILDEMRKKSTQDGLKTTG
         OKS (Plant PKS type III)               KFR--------------------ATRTVLWDCGNMVSACVLYILDEMRRKSADEGLETYG
         CHS (Fungal PKS type III)              ALA--------------------ASNEIYRTRGNTSSVAVLAVL-DKVRELKL-------
         CHS (Fungal PKS type III)              HMR--------------------ASYDTYINHGNSSSATIFSVL-DRLRDADMDRLAVGG
         AggA (Bacterial PKS type III)          ALA--------------------PSRGVLEAYGNCSSATVLLVL-DALRRQED-------
         SrsA (Bacterial PKS type III)          ALD--------------------VTWRSLADVGNLSSSSVLHVLRDTIEQRRPE------
```

Supplementary Figure 13. Sequence alignment of the KS domains in types I and III polyketide synthases (PKSs) and TAS1. KS domain sequences were aligned with MUSCLE, and gaps in the type III PKSs were adjusted to align the catalytic residues. Only the regions including catalytic residues are shown. Catalytic triad residues (orange) and surrounding regions (yellow) are highlighted. TAS1 sequences in the highlighted regions are underlined.

Supplementary Table 1. Microarray data of secondary metabolism-related genes.

Locus Tag	Intensity			Fold change		Putative product name	Description
	WT	Δ*osm1*	WT+DMSO	Δ*osm1* / WT	WT+DMSO / WT		
MGG_00022	527	515	546	1.0	1.0	bassianolide synthetase	NRPS
MGG_00241	1050	1109	1071	1.1	1.0	polyketide synthase	PKS
MGG_00428	3336	3328	3131	1.0	0.9	conidial yellow pigment biosynthesis polyketide synthase	PKS
MGG_00806	2603	4757	4226	1.8	1.6	hypothetical protein	PKS
MGG_01949	987	997	974	1.0	1.0	ent-kaurene synthase	Di-terpene cyclases
MGG_03401	6262	4825	6271	0.8	1.0	hypothetical protein	NRPS
MGG_03810	1693	1472	1429	0.9	0.8	polyketide synthase	PKS-NRPS
MGG_05589	1639	2028	1483	1.2	0.9	lovastatin nonaketide synthase	PKS
MGG_06540	873	845	858	1.0	1.0	tryptophan dimethylallyltransferase 2	DMATS
MGG_07219	3356	6423	2295	1.9	0.7	conidial yellow pigment biosynthesis polyketide synthase	PKS
MGG_07803	877	2213	4312	2.5	4.9	TeA synthetase TAS1	NRPS-PKS
MGG_07858	626	642	648	1.0	1.0	cyclic peptide synthetase	NRPS
MGG_08236	1460	1375	1466	0.9	1.0	polyketide synthase	PKS
MGG_08281	2106	2032	2769	1.0	1.3	polyketide synthase	PKS
MGG_09589	1080	1158	1136	1.1	1.1	polyketide synthase	PKS-NRPS
MGG_09645	1725	1306	1728	0.8	1.0	polyketide synthase	PKS
MGG_10011	1437	1475	1326	1.0	0.9	polyketide synthase	PKS
MGG_10953	1317	1301	1317	1.0	1.0	tryptophan dimethylallyltransferase	DMATS
MGG_11638	1349	1236	1283	0.9	1.0	lovastatin nonaketide synthase	PKS
MGG_12175	2038	2037	1986	1.0	1.0	gramicidin S synthetase 1	NRPS
MGG_12447	884	815	963	0.9	1.1	polyketide synthase/peptide synthetase	PKS-NRPS
MGG_12480	1882	1968	2057	1.0	1.1	hypothetical protein	DMATS
MGG_12613	617	632	637	1.0	1.0	polyketide synthase	PKS
MGG_13767	952	854	943	0.9	1.0	lovastatin nonaketide synthase	PKS
MGG_14722	502	502	504	1.0	1.0	ent-kaurene synthase	Di-terpene cyclases
MGG_14897	1141	1176	1156	1.0	1.0	polyketide synthase	PKS-NRPS
MGG_14943	2079	1979	2061	1.0	1.0	lovastatin nonaketide synthase	PKS-NRPS
MGG_14945	1429	1595	1393	1.1	1.0	polyketide synthase	PKS
MGG_14967	1809	1931	1975	1.1	1.1	tyrocidine synthetase 1	NRPS
MGG_15097	807	838	830	1.0	1.0	polyketide synthase	PKS-NRPS
MGG_15100	909	1002	916	1.1	1.0	polyketide synthase	PKS
MGG_15272	977	1017	946	1.0	1.0	polyketide synthase	PKS

DMATS: Dimethylallyltryptophan Synthase

Supplementary Table 2. Top 30 Basic Local Alignment Search Tool (BLAST) search results for the TAS1 protein.

	Description	Query cover	E value	Identity	Accession	Domain structure	Taxonomy
Fungal source	non-ribosomal peptide synthetase [*Alternaria alternata* ATCC 11680]	99%	0	50%	ATN_PP10953	C-A-PCP-KS	Ascomycota
	non-ribosomal peptide synthetase [*Myceliophthora thermophila* ATCC 42464]	99%	0	49%	XP_003660956.1	C-A-PCP-KS	Ascomycota
	Putative Non-ribosomal peptide synthetase [*Torrubiella hemipterigena*]	99%	0	47%	CEJ93579.1	C-A-PCP-KS	Ascomycota
	non-ribosomal peptide synthetase [*Cordyceps militaris* CM01]	99%	0	47%	XP_006667652.1	C-A-PCP-KS	Ascomycota
	polyketide synthetase [*Gloeophyllum trabeum* ATCC 11539]	98%	0	38%	XP_007868147.1	C-A-PCP-KS	Basidiomycota
	hypothetical protein K443DRAFT_100261 [*Laccaria amethystina* LaAM-08-1]	98%	0	36%	KIK00478.1	C-A-PCP-KS	Basidiomycota
	hypothetical protein GALMADRAFT_130449 [*Galerina marginata* CBS 339.88]	98%	0	36%	KDR67250.1	C-A-PCP-KS	Basidiomycota
	hypothetical protein K443DRAFT_95740 [*Laccaria amethystina* LaAM-08-1]	98%	0	37%	KIK03064.1	C-A-PCP-KS	Basidiomycota
	amino acid adenylation domain protein [*Metarhizium acridum* CQMa 102]	98%	0	37%	XP_007815200.1	C-A-PCP-KS	Ascomycota
	hypothetical protein H633G_01494 [*Metarhizium anisopliae* BRIP 53284]	97%	0	37%	KJK94653.1	C-A-PCP-KS	Ascomycota
	amino acid adenylation domain protein [*Metarhizium guizhouense* ARSEF 977]	97%	0	36%	KID84426.1	C-A-PCP-KS	Ascomycota
	hypothetical protein H634G_03412 [*Metarhizium anisopliae* BRIP 53293]	97%	0	36%	KJK81393.1	C-A-PCP-KS	Ascomycota
	polyketide synthase [*Fusarium avenaceum*]	97%	0	35%	KIL88590.1	C-A-PCP-KS	Ascomycota
	amino acid adenylation domain protein [*Metarhizium anisoplia* e ARSEF 549]	97%	0	36%	KID66114.1	C-A-PCP-KS	Ascomycota
	Polyketide synthase PksJ [*Beauveria bassiana* D1-5]	96%	0	36%	KGQ12647.1	C-A-PCP-KS	Ascomycota
	hypothetical protein FOWG_04281 [*Fusarium oxysporum* f. sp. lycopersici MN25]	97%	0	34%	EWZ97080.1	C-A-PCP-KS	Ascomycota
	amino acid adenylation domain protein [*Beauveria bassiana* ARSEF 2860]	96%	0	36%	XP_008597646.1	C-A-PCP-KS	Ascomycota
	hypothetical protein HIM_00271 [*Hirsutella minnesotensis* 3608]	98%	0	35%	KJZ80421.1	C-A-PCP-KS	Ascomycota
	amino acid adenylation domain protein [*Metarhizium anisopliae*]	88%	0	37%	KFG77666.1	A-PCP-KS	Ascomycota
	hypothetical protein FOYG_11936 [*Fusarium oxysporum* FOSC 3-a]	88%	0	35%	EWY84487.1	C-A-PCP-KS	Ascomycota
	amino acid adenylation domain protein [*Metarhizium brunneum* ARSEF 3297]	86%	0	37%	KID73861.1	PCP-A-PCP-KS	Ascomycota
	hypothetical protein BOTBODRAFT_28419 [*Botryobasidium botryosum* FD-172 SS1]	97%	0	38%	KDQ18939.1	C-A-PCP-PCP-KS	Basidiomycota
	amino acid adenylation domain protein [*Metarhizium majus* ARSEF 297]	80%	0	36%	KID90358.1	C-A-PCP-KS*	Ascomycota
	hypothetical protein FOWG_17753 [*Fusarium oxysporum* f. sp. lycopersici MN25]	81%	0	34%	EWZ77881.1	C-A-PCP-KS*	Ascomycota
	thiotemplate mechanism natural product synthetase [*Grosmannia clavigera* kw1407]	73%	0	35%	EFW99106.1	A-PCP-KS*	Ascomycota
Bacterial source	hypothetical protein [*Mesorhizobium* sp. L2C084A000]	97%	1.00E-158	29%	WP_031213942.1	C-A-PCP-KS-AT-ACP-TE	α-proteobacteria
	hypothetical protein X734_31850 [*Mesorhizobium* sp. L2C084A000]	96%	5.00E-156	29%	ESZ19950.1	C-A-PCP-KS-AT-ACP-TE	α-proteobacteria
	amino acid adenylation domain protein [*Clostridium termitidis* CT1112]	97%	1.00E-152	28%	EMS73654.1	A-PCP-C-A-PCP-KS-AT-KR-ACP	Firmicutes
	thioester reductase [*Clostridium papyrosolvens*]	97%	3.00E-147	27%	WP_004616554.1	A-PCP-C-A-PCP-KS-AT-KR-ACP	Firmicutes
	hypothetical protein X756_24210 [*Mesorhizobium* sp. LSHC412B00]	97%	1.00E-146	28%	ESX84924.1	C-A-PCP-KS-AT-ACP-TE	α-proteobacteria

A BLAST search was conducted at on the amino acid level with whole- length of TAS1 via NCBI and ADG database. ATN_PP10953 was searched from ADG database and all others were NCBI database. Domain structure was predicted with a by polyketide synthase-non-ribosomal peptide synthetase (PKS-/NRPS) analysis web site (http://nrps.igs.umaryland.edu/nrps/) or Pfam (http://pfam.xfam.org/search). KS*, C-terminal truncated KS.

Supplementary Table 3. Strains and plasmids used in this study

Strains and plasmids	Relevant characteristics	Source or reference
Strains		
Magnaporthe oryzae Kita1	Pathogenic to rice plants	Ref. 58
M. oryzae Δosm1	*OSM1 (MGG_01822)* gene disrupted M. oryzae	This study
M. oryzae ΔTAS1	*TAS1* (*MGG_07803*) gene disrupted *M. oryzae*	This study
M. oryzae OE::*TAS1*	Over-expressed *TAS1* gene carried *M. oryzae*	This study
M. oryzae OE::*TAS1ΔKS*	Over-expressed KS domain deleted *TAS1* gene carried *M. oryzae*	This study
S. cerevisiae BJ5464	*MATα ura3-52 his3-Δ200 leu2- Δ1 trp1 pep4::HIS3 prb1 Δ1.6R can1 GAL*	ATCC
E. coli DH5α	F^- φ80d*lacZ* ΔM15Δ(*lacZYA-argF*)*U169 endA1 recA1 hsdR17* ($r_K^- m_K^+$) *deoR thi-1,supE44 gyrA96 relA1* λ^-*phoA*)	Takara
BL21(DE3)pLysS	F^- *ompT hsdS* (r_B^- m_B^-) *gal dcm* λ(DE3) pLysS (Cam^r)	Promega
Plasmids		
pBI121	Binary vector, Km^r, CaMV promoter, β-glucuronidase (GUS)	Clontech
pBI-OSM1::HPH	pBI121 containing up & down stream of *OSM1* and Hyg^r unit between RB and LB	This study
pBI-M07803::HPH	pBI121 containing up & down stream of *MGG_07803* and Hyg^r unit between RB and LB	This study
pBI-OE::TAS1	pBI121 containing up & down stream including *TAS*, BS^r unit, *TEF1* promoter between RB and LB	This study
pBI-TAS1-KS::HPH	pBI121 containing up & down stream including ΔKS-*TAS1*, Hyg^r unit, *TEF1* promoter between RB and LB	This study
pCRII-Blunt TOPO	*Km^r*, *lac* promoter containing plasmid for TOPO cloning	Toyobo
YEp352 ADH1	Amp^r, *ADH1* promoter, *URA3*	Ref. 61
YEp352-MoTAS	YEp352 containing C-terminally 8xhis tagged *TAS*	This study
pESC-Trp	Amp^r, *GAL1/GAL10* divergent promoter, *TRP1*	Stratagene
pESC-Trp-ADH1-MoPPT	pESC-Trp containing ADH1 promoter and *MoPPT1*	This study
pET19b(+)	Amp^r, T7 promoter, *lacI*	Novagen
pET-MoPPT	pET19b containing *MoPPT1*	This study
pEU01	Amp^r, SP6 promoter containing plasmid for cell-free translation	Cell-Free Science
pEU01_C-His_CAT	pEU01 containing C-terminally 8xhis tagged C-A-T domain of *TAS1*	This study
pEU01_N-His_KS	pEU01 containing N-terminally 8xhis tagged KS domain of *TAS1*	This study

Supplementary Table 4. Primers used in this study

Name	Sequences	Description
pBI121-RB	**5′-CAGATTGTCGTTTCCCGCCTTCAGTTT-3′**	For pBI vectors
pBI121-LB	**5′-CGTCCGCAATGTGTTATTAAGTTGTCTAAGC-3′**	For pBI vectors
5HPH	**5′-AAGCTTATCGATACCGTCGACAGAAGATG-3′**	For pBI vectors
3HPH	**5′-CGCGTTTTATTCTTGTTGACATGGAGC-3′**	For pBI vectors
ΔM07803_UP-F	**5′-GGAAACGACAATCTGAGCGAGGTCATGAACAGGGCCAGCAC-3′**	For pBI-TAS1::HPH
ΔM07803_UP-R	**5′-CAAGAATAAAACGCGTGCGACATCACCACGATGGCGAAAG-3′**	For pBI-TAS1::HPH
ΔM07803_DN-F	**5′-GGTATCGATAAGCTTATGGAGTTCCTGCCATACTTGAAAAT-3′**	For pBI-TAS1::HPH
ΔM07803_DN-R	**5′-AACACATTGCGGACGATCGACCAACGGCCCCATTAAATATTA-3′**	For pBI-TAS1::HPH
ΔM07803_CHK_F	**5′-AGCATCAGCTGCCAGTACTGCGTG-3′**	For pBI-TAS1::HPH
ΔM07803_CHK_R	**5′-TCAAAACTAGTAAATCTGGCCTAAG-3′**	For pBI-TAS1::HPH
OETAS1_UP-F	**5′-GGAAACGACAATCTGAGCGAGGTCATGAACAGGGCCAGCAC-3′**	For pBI-OE::TAS1
OETAS1_UP-R	**5′-GGTATCGATAAGCTTGATGATACTGGTACACAGGGTAATGGT-3′**	For pBI-OE::TAS1
AoTEF1-F	**5′-CAAGAATAAAACGCGGACCAGACAGGCGCCACTCGGCCGGGC-3′**	For pBI-OE::TAS1
AoTEF1-R	**5′-GTGCGAACTTTGTAGTTCTTTGTAAGA-3′**	For pBI-OE::TAS1
MoTAS1-F	**5′-CTACAAAGTTCGCACATGAGAGGATCGCATCACCATCACCAT-3′**	For pBI-OE::TAS1
MoTAS1-R	**5′-TCAAAAGAAAGCCTTCTGTTCCACCCCG-3′**	For pBI-OE::TAS1
OETAS1_DN-F	**5′-AAGGCTTTCTTTTGAGCAGGGCGGACTCGGAGCAATATTTTA-3′**	For pBI-OE::TAS1
OETAS1_DN-R	**5′-AACACATTGCGGACGATCGACCAACGGCCCCATTAAATATTA-3′**	For pBI-OE::TAS1
OETAS1_CHK-F	**5′-AGCATCAGCTGCCAGTACTGCGTG-3′**	For pBI-OE::TAS1
OETAS1_CHK-R	**5′-ATCGTGGACGGCCATGTACGACTTTGACAT-3′**	For pBI-OE::TAS1
ΔKS_UP-F	**5′-GGAAACGACAATCTGAGCGAGGTCATGAACAGGGCCAGCAC-3′**	For pBI-TAS1-KS::HPH
ΔKS_UP-R	**5′-GGTATCGATAAGCTTGATGATACTGGTACACAGGGTAATGGT-3′**	For pBI-TAS1-KS::HPH
TEF1+CAT-F	**5′-CAAGAATAAAACGCGGACCAGACAGGCGCCACTCGGCCGGGC-3′**	For pBI-TAS1-KS::HPH
TEF1+CAT-R	**5′-CTTGGGGTTGGGCTCCAGGTTCCTTTCGGCGTCGACAGGAAC-3′**	For pBI-TAS1-KS::HPH
ΔKS_Down-F	**5′-GAGCCCAACCCCAAGTGCGAGTTCGAAGAG-3′**	For pBI-TAS1-KS::HPH
ΔKS_Down-R	**5′-AACACATTGCGGACGATCGACCAACGGCCCCATTAAATATTA-3′**	For pBI-TAS1-KS::HPH
ΔKS_CHK-F	**5′-CCGGCCTCGATCGACTGTGACCGCA-3′**	For pBI-TAS1-KS::HPH
ΔKS_CHK-R	**5′-TCAAAACTAGTAAATCTGGCCTAAG-3′**	For pBI-TAS1-KS::HPH
RACE_GSP-5	**5′-ATCGTGGACGGCCATGTACGACTTTGACAT-3′**	For RACE PCR
RACE_GSP-3	**5′-TCAAGATGGCATTCGACCGCCTCAGCAAGA-3′**	For RACE PCR
TAS1-F	**5′-AACTAAGCTGAATTCATGTCTGTCAGGCCACTACCTTTTCTC-3′**	For YEp352-TAS1
TAS1-R	**5′-GTGGTGGTGGTGGTGAAAGAAAGCCTTCTGTTCCACCCCGAA-3′**	For YEp352-TAS1
YEp352-F	**5′-CACCACCACCACCACCACCACCACTAAAAGCTTTGGACTTCTTCGCCA-3′**	For YEp352-TAS1
YEp352-R	**5′-GAATTCAGCTTAGTTGATTGTATGCTT-3′**	For YEp352-TAS1
MoPPT-F	**5′-AACTAAGCTGAATTCATGAGCTCAGAGTCTAGCCCCGAGATC-3′**	For YEp352-MoPPT
MoPPT-R	**5′-AGAAGTCCAAAGCTTCTACTGCTCTTCTGCGAACTTGACAAT-3′**	For YEp352-MoPPT
ADH1-F	**5′-GGATCCGTGGAATATTTCGGATATCC-3′**	For pESC-Trp-ADH1-MoPPT
ADH1-R	**5′-GACGGATTACAACAGGTATTGTCCTC-3′**	For pESC-Trp-ADH1-MoPPT
pESC-Trp-F	**5′-TATAAGGTGCCTAGGTTCTAGGTCGACGTAATTACTTAGCCG-3′**	For pESC-Trp-ADH1-MoPPT
pESC-Trp-R	**5′-GACAACATTAGGCAGAAGTCGACCGCATTATCGCTTCTCCGG-3′**	For pESC-Trp-ADH1-MoPPT
pET-MoPPT-F	**5′-CATCACCATCACTCCATGAGCTCAGAGTCTAGCCCCGAGATC-3′**	For pET-MoPPT
pET-MoPPT-R	**5′-TTAGCAGCCGGATCCCTACTGCTCTTCTGCGAACTTGACAAT-3′**	For pET-MoPPT
pET-F	**5′-GGATCCGGCTGCTAACAAAGCCCGAA-3′**	For pET-MoPPT
pET-R	**5′-GTGATGGTGATGCGATCCTCTCATGGT-3′**	For pET-MoPPT
pEU01-F	**5′-GTCGACGTCCCATGGTTTTGTAT-3′**	For pEU01_C-His_CAT
pEU01-R	**5′-ACTAGTGATATCTTGGTGATGTAGATAGGTGG-3′**	For pEU01_C-His_CAT
pEU_CAT-F	**5′-CAAGATATCACTAGTATGCATCACCATCACCATCACTCCATGTCTGTCAGGCCACTACC-3′**	For pEU01_C-His_CAT
pEU_CAT-R	**5′-CCATGGGACGTCGACTCATTGCGACCGAGAGCTGCTGCCACC-3′**	For pEU01_C-His_CAT
pEU_KS-F	**5′-CAAGATATCACTAGTATGTCCAGACGGTCGGCAGAAACCAGC-3′**	For pEU01_N-His_KS
pEU_KS-R	**5′-CCATGGGACGTCGACTCAGTGATGGTGATGGTGATGAAAGAAAGCCTTCTGTT-3′**	For pEU01_N-His_KS
ApaI-5’OSM1-F	**5′-GGGCCCGCTGATCTCCTATCCATCAATCG-3′**	For pBI-OSM1::HPH
MluI-5’ OSM1-R	**5′-ACGCGTAGGTTTCAAGGCCTACTGTAAC-3′**	For pBI-OSM1::HPH
XbaI-3’ OSM1-F	**5′-TCTAGAGAAGCAAGGGTTGAGAGCATAC-3′**	For pBI-OSM1::HPH
BsiWI-3’ OSM1-R	**5′-CGTACGACGTTTAAGCAGCAAGACCAAG-3′**	For pBI-OSM1::HPH
OSM1-check-F	**5′-TGTTACAGTAGGCCTTGAAACC-3′**	For pBI-OSM1::HPH
OSM1-check-R	**5′-GTATGCTCTCAACCCTTGCTTC-3′**	For pBI-OSM1::HPH
MluI-HYG-F	**5′-TTTACGCGTAAGCTTATCGATACCGTCGACAG-3′**	For pBI-OSM1::HPH
XbaI-HYG-R	**5′-TTTTCTAGACGCGTTTTATTCTTGTTGAC-3′**	For pBI-OSM1::HPH
ApaI-RB	**5′-TTTTGGGCCCGATTGTCGTTTCCCGCCTTCAG-3′**	For pBI-OSM1::HPH
BsiWI-LB	**5′-TTTTCGTACGTCCGCAATGTGTTATTAAGTTG-3′**	For pBI-OSM1::HPH

Supplementary Methods

OSM1 gene disruption

The PCR primers used are listed in Supplementary Table 4. *OSM1* disruptants were constructed as follows. The upstream sequence of *OSM1* was amplified from the genomic DNA of *M. oryzae* by PCR using ApaI-5′ OSM1-F and MluI-5′ OSM1-R primers. The 1.0 kb fragment was gel purified, TOPO-cloned, digested with *Apa*I and *Mlu*I, and gel purified (fragment 1). The hygromycin B-resistance gene expression unit was amplified from pCSN45 by PCR with MluI-HYG-F and XbaI-HYG-R primers. The 1.6 kb fragment was gel purified, TOPO-cloned, digested with *Mlu*I and *Xba*I, and gel purified (fragment 2). The downstream sequence of *OSM1* was amplified from the genomic DNA of *M. oryzae* by PCR with XbaI-3′ OSM1-F and BsiWI-3′ OSM1-R primers. The 5.0 kb fragment was gel purified, TOPO-cloned, digested with *Xba*I and *Bsi*WI, and gel purified (fragment 3). The vector sequence of pBI121 between the right border and left border was amplified from pBI121 by PCR with ApaI-RB and BsiWI-LB primers. The 8.7 kb fragment was gel purified, TOPO-cloned, digested with *Apa*I and *Bsi*WI, and gel purified (fragment 4). The 4 fragments were ligated to yield a *OSM1* disruption vector, pBI- OSM1::HPH. The *A. tumefaciens* strain transformed with this plasmid was used for ATMT. *OSM1* disruptants were selected by PCR with OSM1-check-F and OSM1-check-R primers, which hybridize upstream and downstream of the deleted *OSM1* ORF, respectively. This primer set can amplify 1.7 kb *OSM1* ORF from wild-type strains, the 1.6-kb hygromycin B-resistance gene expression unit from *OSM1* disruptants, and both fragments from ectopic transformants. The PCR products were digested with *Eco*RV, yielding 0.7 kb and 1.0 kb fragments from the 1.7 kb *OSM1* ORF and an intact 1.6 kb fragment from the hygromycin B-resistance gene expression unit. The Δ*osm1-2* and Δ*osm1-5* strains were selected as *OSM1* gene-disrupted strains (Supplementary Fig. 2).

Rapid amplification of cDNA ends (RACE) PCR

To determine the ORF of TAS1, we conducted 5′ and 3′ RACE PCR with a SMARTer RACE cDNA Amplification kit (Clontech). Total RNA (1 μg) from *M. oryzae* was used for cDNA synthesis, and then 5′ and 3′ RACE PCR was conducted with universal and gene-specific primers (for 5′, RACE_GSP-5; for 3′, RACE_GSP-3) according to the manufacturer's instructions. The RACE PCR product was cloned into pCRII-Blunt TOPO using a TOPO PCR cloning kit (Invitrogen) and then transformed into *E. coli* DH5α. Transformants were selected on an LB plate supplemented with Km (50 μg/ml). The obtained cDNA-inserted plasmid was sequenced.

Artificial intermediate synthesis

To a stirred solution of *N*-(*tert*-butoxycarbonyl)-L-isoleucine (92.4 mg, 0.4 mmol) in tetrahydrofuran (10 ml) was added a mixture of *N*-(3-dimethylaminopropyl)-*N*2-ethylcarbodiimide hydrochloride (76.7 mg, 0.4 mmol) and 1-hydroxybenzotriazole (54.0 mg, 0.4 mmol) in tetrahydrofuran (5 mL), followed by *N*-acetylcysteamine (SNAC; 42.6 μl, 0.4 mmol). After the mixture was stirred for 1 h at room temperature, K_2CO_3 (27. 6 mg, 0.2 mmol) was added, and the resulting mixture was stirred for 3 h at room temperature. The solvent was evaporated under vacuum, and the residue was dissolved in ethyl acetate (20 ml). The organic layer was washed with 10% aqueous $NaHCO_3$ solution and brine, dried over Na_2SO_4, and concentrated. The crude product was purified via SiO_2 column chromatography (*n*-hexane:ethyl acetate = 1:3) to provide Boc-L-Ile-SNAC (118.4 mg, 89%) as a colorless oil.

^{1}H NMR (500 MHz, $CDCl_3$) δ 0.91 (t, *J* = 7.5 Hz, 3H), 0.97 (d, *J* = 7.0 Hz, 3H), 1.13 (m, 1H), 1.42 (m, 1H), 1.47 (s, 9H), 1.96 (s, 3H), 1.98 (m, 1H), 3.04 (m, 2H), 3.39 (ddt, *J* = 13.0, 6.5, 6.5 Hz, 1H), 3.46 (ddt, *J* = 13.0, 6.5, 6.5 Hz, 1H), 4.27 (dd, *J* = 9.0, 5.0 Hz, 1H), 5.00 (d, *J*

= 9.0 Hz, 1H), 5.99 (br, 1H); ^{13}C NMR (125 MHz, $CDCl_3$) δ 11.6, 15.8, 23.1, 24.4, 28.3, 37.4, 39.4, 65.2, 80.4, 155.6, 170.3, 201.9.

Boc-L-Ile-SNAC (111.4 mg, 0.34 mmol) was dissolved in trifluoroacetic acid (0.5 ml) and CH_2Cl_2 (1 ml). The mixture was stirred for 1 h at room temperature and concentrated under vacuum. The residue was twice taken up in CH_2Cl_2 and concentrated to remove the trifluoroacetic acid. The residue was dissolved in CH_2Cl_2 (5 mL) and then triturated by adding diethyl ether (5 ml). The resulting precipitate was filtrated, washed with diethyl ether, and dried to afford L-Ile-SNAC (104.1 mg, 88%).

^1H NMR (500 MHz, CD_3OD) δ 0.99 (t, *J* = 7.0 Hz, 3H), 1.06 (d, *J* = 7.0 Hz, 3H), 1.32 (m, 1H), 1.55 (m, 1H), 1.92 (s, 3H), 2.04 (m, 1H), 1.96 (s, 3H), 3.14 (dt, *J* = 13.0, 6.5, Hz, 1H), 3.19 (dt, *J* = 13.0, 6.5 Hz, 1H), 3.39 (t, *J* = 6.5 Hz, 2H), 4.17 (d, *J* = 4.0 Hz, 1H); ^{13}C NMR (125 MHz, CD_3OD) δ 12.0, 15.0, 22.5, 25.8, 29.8, 38.3, 39.5, 64.9, 118.1 (q, *J* = 293.3 Hz), 162.8 (q, *J* = 33.8 Hz), 173.5, 196.9.

To a stirred solution of L-Ile-SNAC (34.1 mg, 0.1 mmol) in dehydrated ethanol (2 ml) were added sodium ethoxide (6.8 mg, 0.1 mmol) and diketene (7.6 µl three times, 0.3 mmol) at 0~5 °C under N_2. The mixture was stirred for 2 h at room temperature, neutralized with saturated NH_4Cl aqueous solution, and evaporated under vacuum. The residue was dissolved in ethyl acetate, washed with brine, dried over Na_2SO_4, and concentrated. The crude product was purified via SiO_2 column chromatography ($CHCl_3$:MeOH = 10:1) to provide *N*-Acetoacetyl-L-Ile-SNAC (19.0 mg, 61%) as a colorless oil.

^1H NMR (500 MHz, $CDCl_3$) δ 0.93 (t, *J* = 7.5 Hz, 3H), 0.97 (d, *J* = 7.0 Hz, 3H), 1.20 (m, 1H), 1.47 (m, 1H), 1.96 (s, 3H), 2.05 (m, 1H), 2.31 (s, 3H), 3.04 (t, *J* = 6.5 Hz, 1H), 3.42 (dt, *J* = 7.0, 6.5 Hz, 1H), 3.53 (s, 2H), 4.61 (dd, *J* = 8.5, 5.0 Hz, 1H), 5.98 (br, 1H), 7.72 (d, *J* = 8.5 1H); ^{13}C NMR (125 MHz, $CDCl_3$) δ 11.6, 15.9, 23.1, 24.5, 28.5, 31.2, 37.2, 39.3, 48.7, 63.9, 166.0, 170.5, 200.4.
